# Distribution and depth of bottom-simulating reflectors in the Nankai subduction margin

**DOI:** 10.1186/s40623-018-0833-5

**Published:** 2018-04-17

**Authors:** Akihiro Ohde, Hironori Otsuka, Arata Kioka, Juichiro Ashi

**Affiliations:** 1 Atmosphere and Ocean Research Institute, The University of Tokyo, 5-1-5 Kashiwanoha, Kashiwa, Chiba 277-8564 Japan; 2 Graduate School of Frontier Sciences, The University of Tokyo, 5-1-5 Kashiwanoha, Kashiwa, Chiba 277-8564 Japan; 3 Earthquake Research Institute, The University of Tokyo, 1-1-1, Yayoi, Bunkyo-ku, Tokyo, 113-0032 Japan; 4 Institut für Geologie, Universität Innsbruck, Innrain 52f, 6020 Innsbruck, Austria

**Keywords:** Surface heat flow, Bottom-simulating reflector, Methane hydrate, Shallow thermal structure, Nankai Trough

## Abstract

Surface heat flow has been observed to be highly variable in the Nankai subduction margin. This study presents an investigation of local anomalies in surface heat flows on the undulating seafloor in the Nankai subduction margin. We estimate the heat flows from bottom-simulating reflectors (BSRs) marking the lower boundaries of the methane hydrate stability zone and evaluate topographic effects on heat flow via two-dimensional thermal modeling. BSRs have been used to estimate heat flows based on the known stability characteristics of methane hydrates under low-temperature and high-pressure conditions. First, we generate an extensive map of the distribution and subseafloor depths of the BSRs in the Nankai subduction margin. We confirm that BSRs exist at the toe of the accretionary prism and the trough floor of the offshore Tokai region, where BSRs had previously been thought to be absent. Second, we calculate the BSR-derived heat flow and evaluate the associated errors. We conclude that the total uncertainty of the BSR-derived heat flow should be within 25%, considering allowable ranges in the P-wave velocity, which influences the time-to-depth conversion of the BSR position in seismic images, the resultant geothermal gradient, and thermal resistance. Finally, we model a two-dimensional thermal structure by comparing the temperatures at the observed BSR depths with the calculated temperatures at the same depths. The thermal modeling reveals that most local variations in BSR depth over the undulating seafloor can be explained by topographic effects. Those areas that cannot be explained by topographic effects can be mainly attributed to advective fluid flow, regional rapid sedimentation, or erosion. Our spatial distribution of heat flow data provides indispensable basic data for numerical studies of subduction zone modeling to evaluate margin parallel age dependencies of subducting plates.

**Electronic supplementary material:**

The online version of this article (10.1186/s40623-018-0833-5) contains supplementary material, which is available to authorized users.

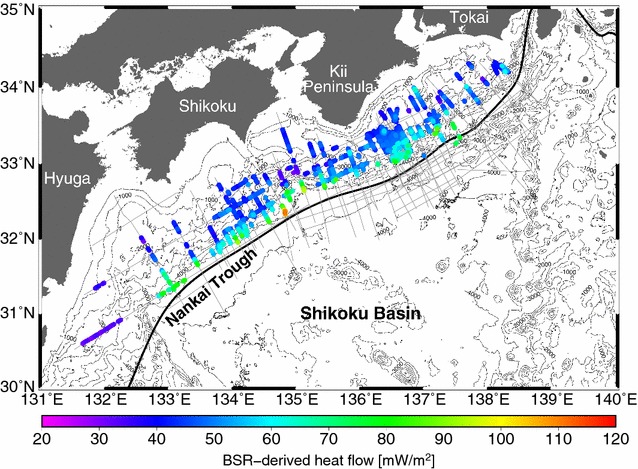

## Introduction

The development of gas hydrates, the guest molecules consisting of almost pure methane (e.g., Kvenvolden 1988), has been confirmed in marine sediments using seismic reflectors, sediment cores, and downhole logging data (e.g., Shipley et al. 1979; Kvenvolden and McDonald 1985; Cook et al. 2010). The range in depth of the methane hydrates has been determined by subseafloor temperatures and pressures (e.g., Shipley et al. 1979; Dickens and Quinby-Hunt 1994). Therefore, the presence of methane hydrate can be used to obtain subseafloor thermal information by taking advantage of the hydrate’s known stability characteristics under low-temperature and high-pressure conditions.

The base of methane hydrates has been generally confirmed from acoustic reflectors called bottom-simulating reflectors (BSRs), which, in seismic reflection images, is characterized by high-amplitude reverse-polarity waveforms paralleling the seafloor (e.g., Markl et al. 1970). Because of the accumulation of methane hydrate above the BSRs (e.g., Stoll et al. 1971) and free gas below the BSRs (Miller et al. 1991; Bangs et al. 1993), BSRs are thought to correspond to the base of gas hydrate stability (BGHS), which is a phase boundary between the methane hydrate and free gas. Therefore, many previous studies have investigated the subseafloor thermal regime based on BSR characteristics (Yamano et al. 1982, 1984; Ashi et al. 2002; Harris et al. 2013). Hyndman and Wang (1993) provided constraints on the seismogenic zone with a thermal model that utilized heat flow values from BSR, probe, and borehole measurements.

The heat flow value is generally dependent on the age of the incoming plate (Stein and Stein 1992). The advection of the subducting plate results in substantially reduced heat flow from the margin wedge and forearc relative to the incoming plate. There are locally high or low heat flow observations superimposed on the regionally low heat flow on the wedge and forearc. These are thought to be mainly due to advective fluid flows (e.g., Wang et al. 1993, 1995), rapid sedimentation or erosion (Hutchison 1985; Wang et al. 1993), or fault activity (e.g., Kinoshita et al. 2011). In addition, topographic effects that arise in convex-upward and convex-downward topographies also cause locally high or low heat flows (Ganguly et al. 2000; Chen et al. 2014; Li et al. 2014). Identifying the phenomena that cause local high or low heat flows is important because surface measurements of heat flow are utilized to estimate thermal properties in plate subduction zones, including frictional strength along plate boundaries and radioactive heat production.

**Fig. 1:**
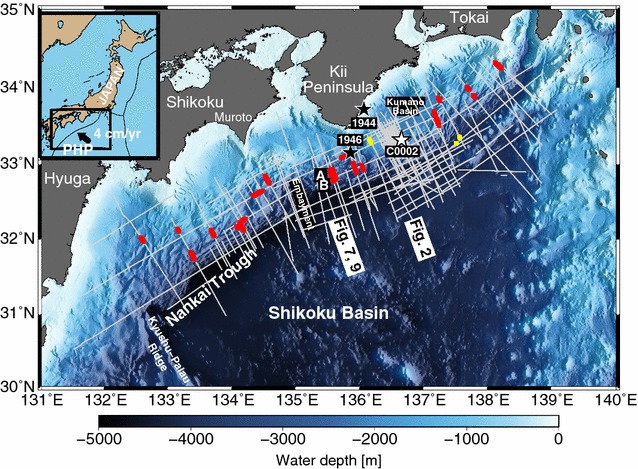
Study area from offshore Tokai to Hyuga in the Nankai subduction margin. Gray lines represent the MCS reflection surveys studied in this work. Yellow dots represent the locations of BSRs identified in Figs. 2, 6b, c. Red dots denote the locations used to calculate the thermal structure via thermal modeling, which adopts the temperatures at the BSR depths as a constraint and, therefore, requires continuous BSRs (see Additional file 1: Figures S2 and S3 for more detail on the modeling section, and Figures S4–S23 for modeling results). Black stars indicate the epicenters of the Tonankai earthquake in 1944 and Nankai earthquake in 1946 (Kanamori 1972). Site C0002 of the IODP Expeditions (Expedition 314 Scientists 2009) is indicated by a white star. Note that there are many other IODP wells where heat flows have been determined using borehole measurements (e.g., Harris et al. 2011; Marcaillou et al. 2012; Harris et al. 2013) (see Fig. 8a). The seismic profile of the A–B section is illustrated in Fig. 7, and the thermal structure modeled over the section is shown in Fig. 9. The parameters used in the thermal modeling of the A–B section are shown in Table 2. A total of 142 profiles are used in this study (see main text for properties of the studied MCS data)

In the Nankai accretionary margin, BSRs are widely distributed through the accretionary prism and forearc basins (Aoki et al. 1982; Ashi et al. 2002; Baba and Yamada 2004; Otsuka et al. 2015). Therefore, heat flow can be acquired by thermistor probes and borehole measurements and by BSRs, which provide continuous heat flow profiles, unlike the instrumental measurements. In this study, we first investigate in detail the distribution of BSRs over the accretionary prism slope and the forearc basins, from offshore Tokai to Hyuga in the Nankai subduction margin (Fig. 1), using two-dimensional (2-D) multi-channel seismic (MCS) reflection data. We also compare the heat flow derived from BSRs with those estimated from probe and borehole measurements. Second, we estimate two-dimensional shallow thermal structures in the Nankai subduction margin that are constrained by temperatures at BSRs. This enables the discussion of factors that influence local BSR depth anomalies in convex and concave topographies, which is the main purpose of our study. This method advances the understanding of the effects of past surface geological phenomena, such as prominent sedimentation or erosion.

## Regional setting

### Geological setting

The Nankai Trough, formed by the northwestward subduction of the Philippine Sea Plate (PHP in Fig. 1) beneath the Eurasian plate at approximately 4 cm/year (e.g., Seno et al. 1993), is a plate boundary where earthquakes of magnitude 8 have occurred at intervals of 100–200 years (e.g., Satake 2015). Recent megathrust earthquakes with magnitude classes of 8 in the Nankai subduction zone include the Tonankai earthquake in 1944 and the Nankai earthquake in 1946 (e.g., Kanamori 1972). The geological structure in the Nankai accretionary margin can be divided from the southeast to the northwest into a deformation front, a prism slope with slope basins, an outer ridge associated with a megasplay fault, and a forearc basin. The Kumano Basin, one of the major forearc basins along the Nankai Trough, is approximately 100 km from east to west and approximately 70 km from north to south (Morita et al. 2004). The Nankai Trough is characterized by a shallow trench due to the subduction of the young plate and contains thick trench-fill sediments at a water depth of approximately 4000 m ranging from south of Suruga Bay to the northern tip of the Kyushu-Palau Ridge.

### Previous heat flow studies

Many previous studies estimated heat flow values using probes and BSRs in the Nankai Trough (e.g., Yamano et al. 1982). Heat flow values gradually decreased from the trough floor to the forearc basin, as Yamano et al. (2003) estimated through a heat flow probe. Although the heat flow values tended to decrease landward, as reported above, they were also found to vary with topography on a local scale, especially in convex-upward and convex-downward seafloor regions, as indicated by Ganguly et al. (2000), who concluded that heat flow could increase by as much as 50% on an undulating seafloor. Harris et al. (2013) found broad agreement between the heat flow measured by probes and those derived from BSRs in the Nankai margin. Offshore Muroto (offshore southeast of Shikoku), high heat flow values of approximately 200 mW/m^2^ were observed by probes in previous studies (e.g., Yamano et al. 2003) and were thought to be caused by vigorous hydrothermal circulation within aquifers in the oceanic crust (Spinelli and Wang 2008; Harris et al. 2013, 2017).

## Seismic data and BSR picking

The data for this study were derived from sixteen MCS reflection survey cruises by the research vessels (R/Vs) *Kairei* and *Kaiyo* (JAMSTEC, Japan), and the R/V *Polar Princess* (GC Rieber Shipping, Norway), for a total of 142 survey lines (Fig. 1). The seismic surveys referred to as KR9702 (acquired in 1997), KR9704 (1997), KR9806 (1998), KR9810 (1998), KR9904 (1999), KR0108 (2001), KR0114 (2001), KR0211 (2002), KR0413 (2004), KR0512 (2005), KR1011 (2010), KR1109 (2011), and KR1212 (2012) were conducted by the R/V *Kairei*; KY0314 (2003) and KY1311 (2013) were conducted by the R/V *Kaiyo*; and ODKMPP03 (2003) was conducted by the R/V *Polar Princess*. Data acquisitions from the KR9702, KR9704, KR9806, and KR9810 surveys were performed with an airgun array with a volume of ~ 66 L fired at 50-m intervals and a streamer with a length of ~ 3.5 km containing 120 receivers. Data from KR9904, KR0108, KR0114, KR0211, and KY0314 were acquired with an airgun array with a volume of ~ 200 L fired at 50-m intervals and a streamer with a length of ~ 4 km containing 156 receivers. Data from KR0413 and KR0512 were obtained with an airgun array with a volume of ~ 200 L fired at 50-m intervals and a streamer with a length of ~ 5.5 km containing 204 receivers. Data from KR1011, KR1109, KR1212, and KY1311 were obtained with an airgun array with a volume of ~ 128 L fired at 50-m intervals and a streamer with a length of ~ 6 km containing 444 receivers. Data from ODKMPP03 were acquired with an airgun array with a volume of ~ 70 L fired at 50-m intervals and a streamer with a length of ~ 6 km containing 480 receivers.

**Fig. 2:**
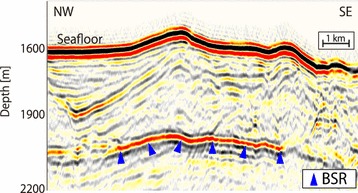
Example of BSR picking from the seismic profile generated by the KR0211 cruise. The picked BSRs are marked with blue triangles; at these points, the seismic profiles exhibited the characteristic of high-amplitude reverse-polarity waveforms paralleling the seafloor compared with the seafloor reflector waveforms. The two-way travel time is converted to subseafloor depth using a P-wave velocity obtained from Site C0002 of the IODP Expeditions (Expedition 314 Scientists 2009)

All the MCS reflection data were processed conventionally with trace editing, common mid-point (CMP) sorting (with CMP intervals of 12.5 m for the seismic data acquired by the R/V *Kairei* and *Kaiyo* cruises completed by 2005; 6.25 m for the data from the ODKMPP03, KR1011, KR1109, and KR1212 surveys; and 3.125 m for the data from the KY1311 survey), band-pass filtering, gain, deconvolution, muting, velocity analysis, normal moveout correction, CMP stacking, and post-stack time migration. The vertical resolution (Rayleigh’s criterion) within the dominant frequencies and studied time domains was 10–20 m. All processing, including imaging and BSR picking, was conducted using the commercial software, Paradigm Product Manager. In this study, the BSRs were carefully identified as distinct acoustic reflectors that displayed the characteristics of paralleling the seafloor based on seismic reflection images that exhibited high-amplitude reverse-polarity waveforms (Fig. 2).

## Methods

### Heat flow derived from BSR depth

Heat flow is generally measured through penetration of a 4.5–6.0-m probe with temperature sensors and occasionally via in situ borehole temperature measurements in deep-sea drilling. However, BSR depths constrained by the pressure–temperature conditions have also been used as an effective method for heat flow estimation (e.g., Yamano et al. 1982). Both the probe and BSR methods have advantages and disadvantages that are related to the regions of interest. In general, the probe method can measure heat flow values at any location where the probe can penetrate the seafloor, whereas the BSR method is restricted to areas where BSRs are present, such as continental margins. In areas of bottom water temperature fluctuation, short thermal probes are more susceptible to uncertainties arising from these variations (Davis et al. 2003), unless the effect is removed through long-term observations of the bottom water temperature (Hamamoto et al. 2005). In contrast, values estimated from the BSRs are less affected by the bottom water temperature variations because, in most areas, the BSR depths are located hundreds of meters below the seafloor. Therefore, the values calculated from the BSRs can be used to estimate the geothermal regime up to approximately 1 km below the seafloor. Moreover, because the presence of BSRs in the Nankai subduction zone has been widely confirmed (Ashi et al. 2002; Baba and Yamada 2004; Otsuka et al. 2015), heat flow values can be calculated over wide areas when physical properties, such as seismic velocity, density, and thermal resistance, are estimated from seismic surveys and deep-sea drilling.

In this study, we used the relationship between the two-way travel time and the depth obtained from the check shot survey at Site C0002 of the Integrated Ocean Drilling Program (IODP) Expedition 314 (Expedition 314 Scientists 2009) to convert the two-way travel time into depth in all MCS reflection images. The IODP Site C0002 was selected because many of our survey lines were densely distributed near IODP Site C0002 (Fig. 1) and because this site afforded the most reliable P-wave velocity, including uncertainty, although some of our MCS reflection images were obtained farther away from the IODP site. The velocity in the overlying seawater column was assumed to be 1500 m/s.

We assumed hydrostatic pressure within the sediment columns above the BSRs; the differences in the results that arise from formation pressures between hydrostatic and lithostatic conditions were discussed in the following section. Using a phase diagram for methane hydrate (Tishchenko et al. 2005), the estimated pressure indicated the temperature at the BSR depth. Although other gases such as CO_2_ and H_2_S affect the phase boundary of methane hydrate (Claypool and Kaplan 1974), core samples revealed that methane hydrate in the Nankai subduction zone consists of almost pure methane (~ 99%) (Tobin et al. 2015).

The seafloor temperature was derived from the hydrographic data of the bottom water temperature from the Japan Oceanographic Data Center, which was then used in part to compute the geothermal gradient. The thermal conductivity of the formation between the seafloor and the BSR was estimated from shipboard measurements of discrete samples obtained at Site C0002 of IODP Expeditions 315 and 338 (Expedition 315 Scientists 2009; Strasser et al. 2014). Values of thermal conductivity corresponding to the subseafloor depth were adopted. As the burial depth increased, sediment compaction occurs and porosity decreased. Consequently, the P-wave velocity and thermal conductivity increased with increasing burial depth. Therefore, integrated values of thermal resistance were adopted at depths between the seafloor and the BSR. Finally, the heat flow was calculated from the geothermal gradient and the thermal resistance.

### Uncertainty in BSR-derived heat flow

When we converted the two-way travel time into depth and calculated thermal resistance, we applied the physical properties from the sedimentary rocks at Site C0002 of the IODP Expeditions (Kinoshita et al. 2009) to the other BSR areas, allowing the heat flow values to be calculated wherever BSRs are recognized. However, errors may become large in cases where the physical properties of the sediment deviate greatly from those of the IODP Site C0002 (Kinoshita et al. 2009).

Determining heat flow from the BSRs involved trade-off relationships among several parameters (e.g., Grevemeyer and Villinger 2001) as discussed below. In this study, we conducted an error evaluation of the heat flow values by varying the P-wave velocity between 1700 and 1900 m/s based on the relationship between the two-way travel time and the depth obtained from IODP Site C0002 (Expedition 314 Scientists 2009) (Table 1) to account for this significant variation in the velocity in the sediment with the presence of different gases or porosities. When we overestimated the P-wave velocity within the sequence above the BSR, the apparent BSR depths appeared deeper in the seismic reflection image, decreasing the geothermal gradient and the thermal resistance. Ultimately, a trade-off between underestimating the geothermal gradient and the thermal resistance reduced the resulting heat flow variation. Moreover, we considered that the heat flow values were calculated using the physical properties of the forearc basin at IODP Site C0002, which may differ from those of the prism slope. Therefore, we also evaluated the uncertainty using the physical properties of the prism slope at Site 1178 of Ocean Drilling Program (ODP) 190 (Moore et al. 2001).

**Table 1:** Results of error evaluation

Profile	WD (m)	*V*_p_ (m/s)	HF (mW/m^2^)
KR9904-7	4472	1700	108
		1800	102
		1900	97
KR9904-8	765	1450 (in seawater)	38
	792	1500 (in seawater)	40
	818	1550 (in seawater)	41

Profiles KR9904-7 and KR9904-8 are derived from offshore of Shikoku

WD, water depth; *V*_p_, P-wave velocity; HF, heat flow derived from the BSRs

Heat flow values calculated from BSRs have large uncertainties reaching up to 20–25% (Townend 1997; Ganguly et al. 2000; Henrys et al. 2003; Marcaillou et al. 2006; Kinoshita et al. 2011). Grevemeyer and Villinger (2001) concluded that uncertainties in heat flow from BSRs fall within 5–10% if probe measurement data can constrain the temperature at BSR depths, while estimated uncertainties can reach 50–60% if probe data are absent. This large error can be produced by picking error in seismic reflection images (Marcaillou et al. 2006) and by the discrepancy between the true value and the adopted value of the P-wave velocity, as well as the thermal conductivity of the sediment (Ganguly et al. 2000; Henrys et al. 2003). Neither double BSRs (Foucher et al. 2002; Chhun et al. 2017) nor foldback reflectors (Otsuka et al. 2015) were found in the survey lines used for our study, preventing errors in the calculation of the heat flow from the BSRs due to misinterpretation of the BSRs.

Our error evaluation showed that the heat flow value reached its maximum when the average P-wave velocity at depths between the seafloor and the BSR was set at its lowest value (Table 1). This could be easily explained by the fact that the BSR depth below the seafloor becomes shallower after the time-to-depth conversion when adopting the low P-wave velocity and because of the associated increase in geothermal gradient. Although both the thermal resistance and the P-wave velocity changed with sediment consolidation, the resultant heat flow was found to be influenced more by the change in geothermal gradient due to P-wave velocity variation than by the change in thermal resistance.

In the same manner, the heat flow exhibited a minimum value when the average P-wave velocity was set at its highest value. Because the wide range of P-wave velocities (1700–1900 m/s) ensured an appropriate error evaluation, the error of the heat flow produced by the maximum and minimum P-wave velocities must be within 11%. The different physical properties of the forearc basin at IODP Site C0002 and the prism slope at ODP Site 1178 caused an average error of 5.2%.

In addition to the error associated with the P-wave velocity of the sediments, the P-wave velocity in the seawater column was affected by temperature and salinity (e.g., Wagner and Pruß 2002; Feistel 2008). However, we considered a P-wave velocity variation of 1450–1550 m/s in the seawater column and confirmed that the heat flow values change by less than 3.9% in the studied area (Table 1). Considering the vertical resolution of the seismic reflection images (< 20 m), the BSR picking also produced an error of no more than 1%. Therefore, the total heat flow uncertainty from the BSR caused by the changes in the P-wave velocity should fall within 25% (± 12.5%) of the value presented in this study.

**Fig. 3:**
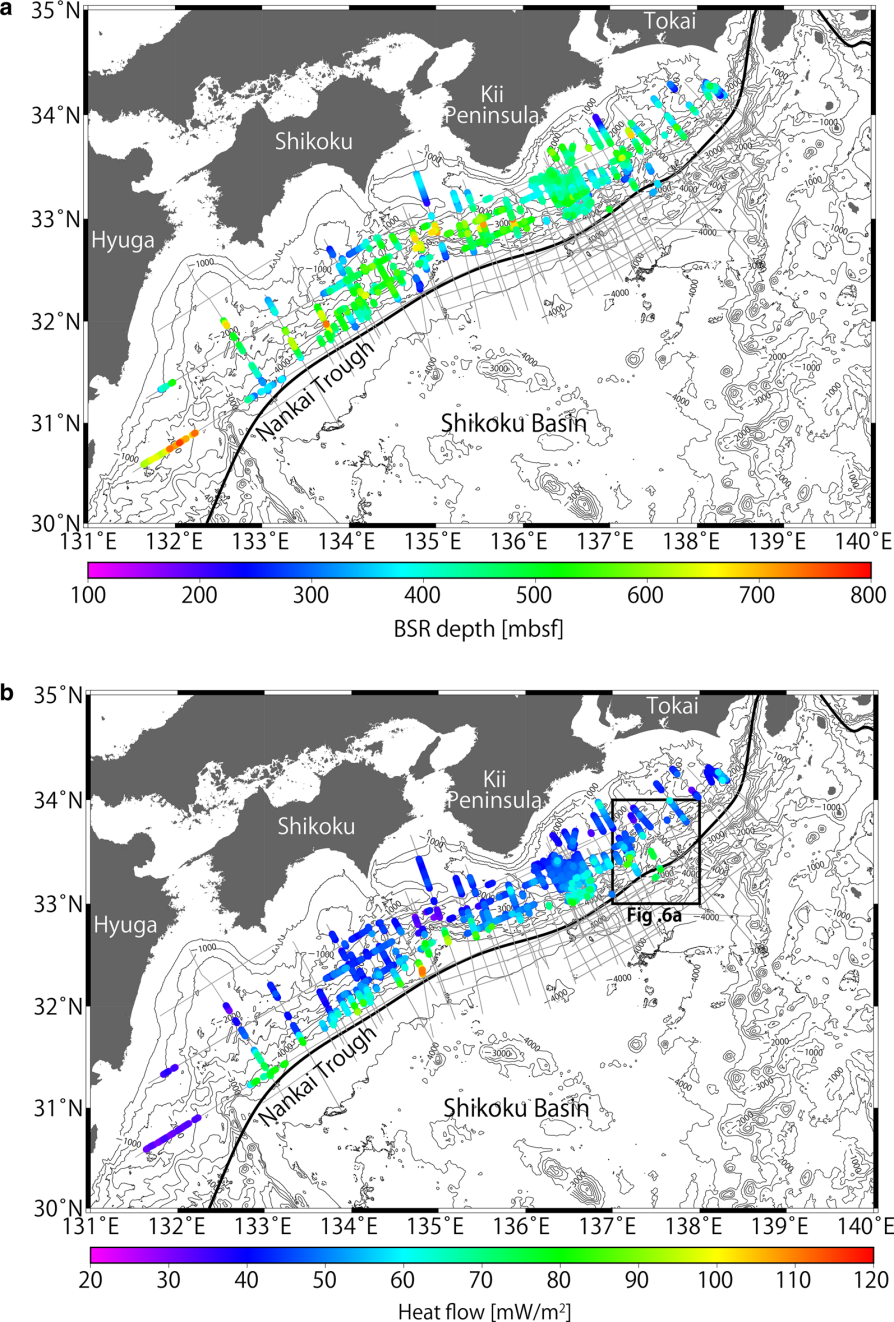
Distributions of methane hydrate BSRs in the Nankai subduction zone. **a** Colored dots indicate BSR depths below the seafloor. Survey lines are represented by gray lines. **b** Colored dots indicate the topographically corrected and uncorrected heat flow derived from the BSRs in this study. Locations of topographically corrected sections are denoted in Fig. 1. Studied MCS profiles are represented by gray lines

**Fig. 4:**
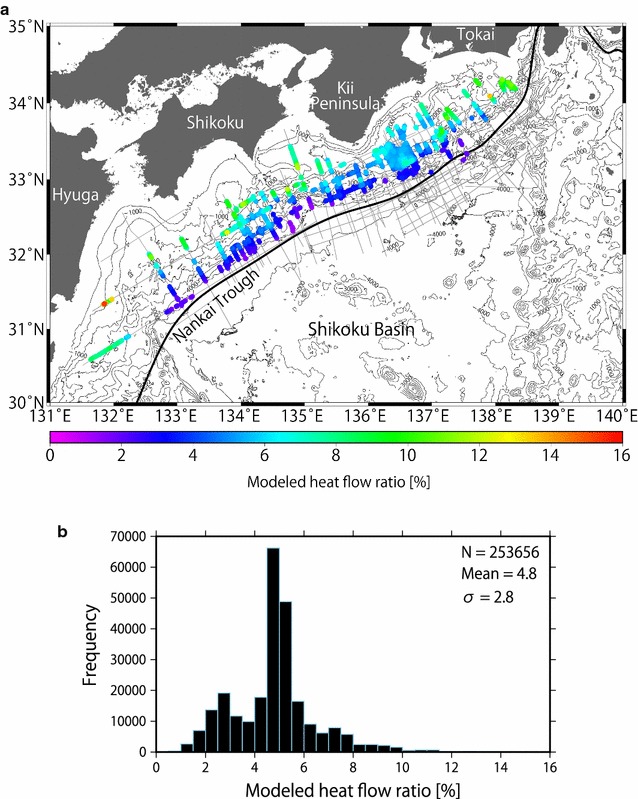
Heat flow differences (HF_l_ − HF_h_)/HF_h_× 100 assuming hydrostatic and lithostatic pressure profiles, respectively. **a** Ratios in the study area. Large values are observed in the shallow water area and small values are distributed in the deep water area. **b** Histogram showing the difference between the heat flows based on hydrostatic pressure and lithostatic pressure. The results are compiled from 142 seismic profiles (253,656 points). The mean ratio and the standard deviation are 4.8 and 2.8, respectively

Furthermore, we calculated the temperatures at the BSRs by assuming the hydrostatic condition in the sedimentary sequence above the BSR. Most previous studies also assumed hydrostatic pressures (e.g., Marcaillou et al. 2012; Li et al. 2014), although intermediate values between hydrostatic and lithostatic pressures were adopted by Ashi et al. (2002). In order to examine the uncertainty caused by these pressure profiles, we calculated heat flow values, HF_h_ (Fig. 3b) and HF_l_ (Additional file 1: Figure S1), from the BSRs using hydrostatic and lithostatic pressures, respectively, and assessed their differences (HF_l_ − HF_h_)/HF_h_× 100 (Fig. 4a). We found the ratio (HF_l_ − HF_h_)/HF_h_× 100 changed by approximately 4.8% on average (Fig. 4b). The ratio had a consistently larger value in shallower areas, while the ratio was smaller in deeper waters, owing to the depth between the seafloor and the BSR with respect to the thickness of the overlying water column. In addition, convex-upward seafloor regions yielded a large ratio because the water depths were slightly shallower allowing deeper BSRs to develop in this location relative to neighboring areas. The ratio in shallow waters (e.g., at a water depth of 800 m) differed up to approximately 15%.

We also compared the BSR-derived values obtained by assuming hydrostatic and lithostatic pressure regimes to the heat flow HF_APCT3_ from an advanced piston corer temperature tool (APCT3) that penetrated 159 m below the seafloor (mbsf) at Site C0002 of IODP Expedition 315 (Expedition 315 Scientists 2009; Harris et al. 2013) and the heat flow HF_probe_ from the probe used at the Kumano Basin near Site C0002 (Hamamoto et al. 2011). The difference (HF_h_ − HF_APCT3_)/HF_APCT3_× 100 fell within 12%, and the difference (HF_l_ − HF_APCT3_)/HF_APCT3_× 100 reached approximately 18%. Because the heat flow comparison may reflect different thermal conductivities, we also calculated the difference using the geothermal gradient in the same manner as the heat flow and obtained values of − 1.9 and + 2.9%, assuming hydrostatic and lithostatic pressures, respectively. In contrast, the difference (HF_h_ − HF_probe_)/HF_probe_× 100 reached approximately − 16%, and the difference (HF_l_ − HF_probe_)/HF_probe_× 100 reached approximately − 10%. The minimum difference was the absolute difference between the geothermal gradient value assuming hydrostatic pressure and the APCT3 geothermal gradient. Because the penetration depth of the APCT3 was much deeper than that of the probe, differences between the BSR-derived heat flow and heat flow obtained with the APCT3 were more reliable. Therefore, we constrained the thermal structure using the temperature at the BSRs by assuming hydrostatic pressure.

### Correction for influence of topography

Surface heat flow values are sensitive to seafloor topography. This topographic effect was first considered in the Cascadia margin (Ganguly et al. 2000; Hornbach et al. 2012; Phrampus et al. 2017), the Costa Rica margin (Harris et al. 2010), and the Nankai Trough (Harris et al. 2011). Li et al. (2014) suggested that three-dimensional topographic effects should be taken into account to evaluate heat flow variations in the complex topographic region of Cucumber Ridge off Vancouver Island, where the dip angle locally reaches ~ 45°. Variations in heat flow can also be explained by the magnitude and pattern of two-dimensional topographic effects, although the difference between the BSR-derived heat flow and the modeled heat flow reached 20 mW/m^2^ over the steep slope of Cucumber Ridge (Li et al. 2014). Because our modeling sections had gentler slopes falling within 20° in the dip direction and relatively little topographic variation, within 5° (3° on average) across the modeling sections, our calculation, which considers two-dimensional topographic effects based on Blackwell et al. (1980), was also considered to provide a reasonable model for shallow thermal structures.

**Fig. 5:**
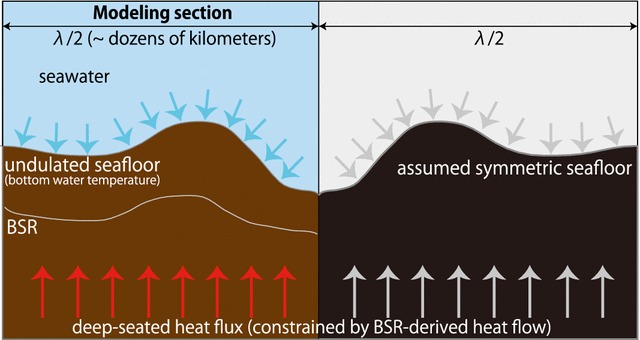
Schematic illustration of the thermal model. Note that this model modified Blackwell et al.’s (1980) protocol by adopting the temperature at BSR depths as the constraint

The topographic effect was calculated by considering the bottom water temperature and the relief of the seafloor (Fig. 5). Although topographic effects were also affected by lithology, the shallow thermal structure in this study was modeled mainly at the prism slope, composed predominantly of mudstone and sandstone (Moore et al. 2001; Strasser et al. 2014); therefore, we ignored the regional lithological variation. In addition, we did not need to consider the influence of seasonal changes in the bottom water temperature on the BSR depth because thermal diffusion from the seafloor to greater depths takes far longer than the seasonal changes (Martin et al. 2004). Therefore, BSR depths could be reliable indices of temperature below the seafloor.

In order to obtain the shallow thermal structure below the seafloor, we solved the following two-dimensional steady-state equation:

1 $$\frac{{\partial^{2} T}}{{\partial x^{2} }} + \frac{{\partial^{2} T}}{{\partial z^{2} }} = 0 ,$$

where $T$ is the subseafloor temperature, $x$ is the horizontal coordinate, and $z$ is the vertical coordinate. The following were employed as boundary conditions: (a) the bottom water temperature at the seafloor from hydrographic observation data from the Japan Oceanographic Data Center as an upper boundary condition; and (b) a geothermal gradient $\alpha$ at infinitely deep depths in the absence of a topographic effect. This method modified Blackwell et al.’s (1980) protocol by using temperatures at observed BSR depths to constrain the boundary condition (b), as detailed below. We also assumed that the thermal conductivity was uniform and that the heat flow from the deep depths was constant and vertical.

The solution of the heat equation, Eq. (1), constrained by these boundary conditions, was obtained by the following series approximation:

2 $$T\left( {x,z} \right) = \alpha z + \mathop \sum \limits_{k = 0}^{M} A_{k} { \exp }\left( { - 2\pi k\frac{z}{\lambda }} \right){ \cos }\left( {2\pi k\frac{x}{\lambda }} \right) ,$$

where $A_{k}$ and $k$ are a constant and a wave number, respectively, determined by the boundary conditions. To simplify the calculation, we assumed that the seafloor depth was symmetric from 0 to $\lambda$, where $\lambda$ is the wavelength of Eq. (2). The wavelength $\lambda$ was twice as long as the horizontal distance because we assumed a symmetric geometry of the seafloor on the lateral boundaries of the region of interest (Fig. 5). Here, $N$ points of discrete topographic data with horizontal intervals corresponding to the CMP interval were present from 0 to $\lambda /2$, and we took $M$ ($< N - 1$) points of the series, where $M$ was a free parameter. The number of discrete topographic data corresponding to the CMP number varied with each survey line (e.g., $N = 1268$ in the A–B section of Fig. 1). We used a free parameter $M$ of 80 because this value was smaller than the number from the topographic data of all modeling survey lines and an increase in $M$ (e.g., from 80 to 1000 in the A–B section of Fig. 1) had little effect on the thermal structure (0.002 °C on average). The coefficient $A_{k}$ could be obtained by solving the square matrix of $M + 1$.

We then acquired temperatures at discretized depths using the obtained coefficient, where the vertical interval was 3–15 m, depending on the horizontal distance. There were 900 vertical components for each horizontal point. The values obtained using Eq. (2) were referred to as topographically corrected values in this study. The parameter values used for thermal modeling in the A–B section of Fig. 1 are shown in Table 2. The temperatures at the BSR depths were used for the boundary condition (b) to restrict the calculated thermal structure by choosing the best coherency between the temperature at the BSRs and the calculated temperature at the same depth of the shallow thermal structure. In particular, the geothermal gradient $\alpha$ must be known before the calculation, and we found the most suitable $\alpha$ in each modeling section. The $\alpha$ value was determined so that the calculated temperature at BSR depths best agreed with the temperature estimated based on the phase boundary. Therefore, we calculated the thermal structure iteratively in order to derive the most suitable geothermal gradient $\alpha$ at intervals of 1 °C/km. The thermal structure was then finalized using the derived geothermal gradient $\alpha$. We conducted these procedures in each modeling section; therefore, the geothermal gradient $\alpha$ differed in each modeling section. The locations of all modeling sections are indicated in Additional file 1: Figures S2 and S3.

**Table 2:** Parameters used for thermal modeling in the A–B cross section southwest of the Kii Peninsula

Parameter	Value
Thermal conductivity	1.0 W/m/K
Free parameter, *M*	80
Number of topographic data, *N*	1268
Wavelength, *λ*	31,700 m

The location of the modeling section is indicated in Fig. 1

## Results

### Distribution of BSRs

**Fig. 6:**
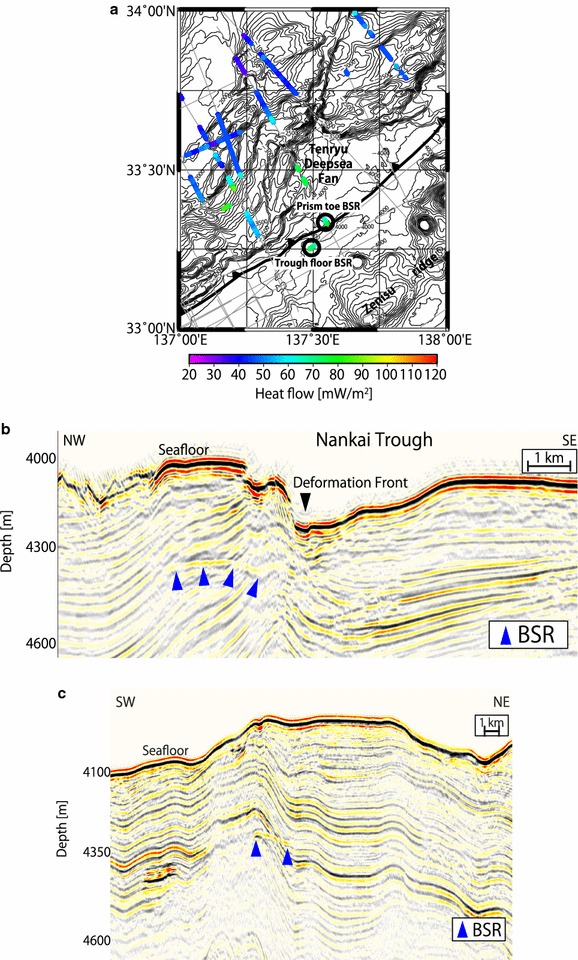
Detected BSRs. **a** Map of BSR-derived heat flow in the Tokai area. BSRs were detected within the locations marked by the two black circles. Colored dots indicate heat flow values derived from the BSRs. **b** Seismic reflection image at the prism toe, where BSRs were found, shown with blue triangles. A P-wave velocity obtained from Site C0002 of the IODP Expeditions (Expedition 314 Scientists 2009) is used for time-to-depth conversion. **c** Seismic reflection image at the trough floor, where BSRs were found, shown with blue triangles. A P-wave velocity obtained from Site C0002 of the IODP Expeditions (Expedition 314 Scientists 2009) is used for time-to-depth conversion

We confirmed the existence of extensive BSRs in the Nankai Trough (Fig. 3), ranging from the forearc basins to the prism slope, which was consistent with previous studies (Ashi et al. 2002; Baba and Yamada 2004). BSRs had been thought to be absent from the prism toe off Tokai as well as the trough floor, slope basins, steep slope, and submarine canyons. However, this study revealed the development of acoustic reflectors that parallel the seafloor with seismic reflection profiles that show high-amplitude reverse-polarity waveforms at the toe and at the trough (trench) floor of the eastern edge of the Nankai subduction margin (Fig. 6), as found in the Costa Rica erosional margin (Pecher et al. 1998). The reflector at the prism toe extended from approximately 1–2 km landward of the trough floor at a water depth of approximately 4000 m. The depth between the seafloor and the reflector was 300–400 mbsf. Similarly, the reflector at the trough floor extended approximately 1 km at a water depth of approximately 4000 m. The depth between the seafloor and the reflector was 300–370 mbsf. We interpreted these acoustic reflectors as BSRs, as further explored in the “Discussion” section.

BSR depths are controlled by in situ temperatures and pressures, and a gas supply to the formation is required for the development of BSRs. Although the trough floor and the prism toe were theoretically deep enough for BSRs to develop, BSRs were generally not observed in these regions, even at great water depths. This suggested a lack of methane in these regions, owing to young horizontal trench sediments consisting of alternating permeable and impermeable layers (e.g., Ashi et al. 2002). In addition, picking BSRs that parallel the reflectors of sedimentary layers was technically challenging with low-amplitude reflectors.

**Fig. 7:**
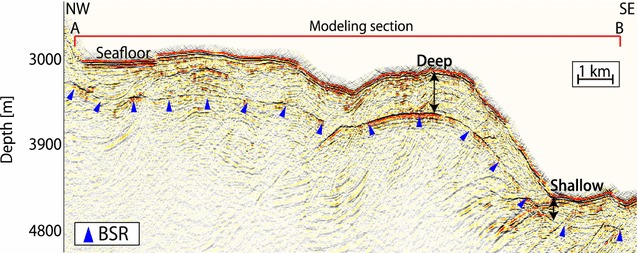
Example of BSR depth variation in convex-upward and convex-downward seafloor regions southwest of Kii Peninsula. BSRs are marked with blue triangles. Their depths are deeper in the convex-upward seafloor region and shallower in the convex-downward seafloor region relative to the neighboring areas. The two-way travel time is converted to subseafloor depth using a P-wave velocity obtained from Site C0002 of the IODP Expeditions (Expedition 314 Scientists 2009)

The depth between the seafloor and the BSR fluctuated between 150 and 800 m in the studied area (Fig. 3a), and uncertainties in the BSR depth were ± 5% considering variation in P-wave velocity, as discussed in the Methods section. A trend of increasing BSR depths from the trough floor to the forearc basin was observed, as reported in previous studies over the prism slope to the forearc basin (Yamano et al. 1982, 1984). Because the BGHS should increase with water depth under a constant heat flow, the observed trend of BSR depth implied that heat flow decreases from the trough floor to the forearc basin. We also observed that the BSR depth was deeper in convex-upward seafloor regions of approximately 120 m and was shallower in convex-downward seafloor regions of approximately 250 m than in neighboring areas (Fig. 7).

### Heat flow estimation

**Fig. 8:**
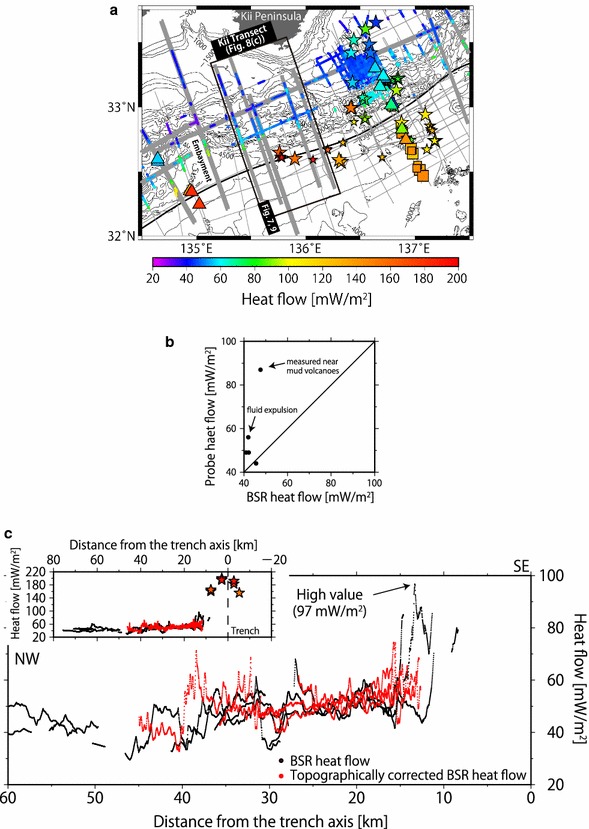
BSR-derived heat flows. **a** Comparison of heat flows from the BSRs, probe measurements, and borehole measurements off the Kii Peninsula. Colored symbols indicate heat flows measured by probes [squares from Kinoshita et al. (2008), stars from Hamamoto et al. (2011)], boreholes [triangles from Shipboard Scientific Party (1975, 1986a, b), Taira et al. (1991), Moore et al. (2001), Harris et al. (2011), and Marcaillou et al. (2012)], and BSRs (colored dots from this study). Larger sizes of the square and star symbols reflect better data quality as defined by the authors. Gray lines represent survey lines, and thick lines represent modeled survey lines. The black frame bounds a 60 km wide area encompassing five modeling sections and probe data. **b** Cross plot showing the topographically uncorrected BSR-derived heat flow and probe heat flow within approximately 1 km of the Kumano Basin. The differences between the heat flows from BSRs and from probes in neighboring areas fall within a range of 20%, except at two sites, which are thought to be affected by fluid expulsion (Hamamoto et al. 2011). **c** Heat flow profiles along the transect bounded by the black frame in (**a**). The profiles include topographically uncorrected (black dots) and corrected (red dots) BSR-derived heat flows of five modeled seismic lines (gray thick lines within the black frame in (**a**). Although a sudden rise/drop of the heat flow is observed in the uncorrected data at distances from the trench axis of approximately 15 and 30 km, the change is tempered by topographic correction. The inset figure shows the topographically uncorrected (black dots) and corrected (red dots) BSR-derived heat flows of the five modeled seismic lines and probe heat flows [stars from Hamamoto et al. (2011)] from the black frame of (**a**)

A maximum heat flow from the BSR of 111 ± 14 mW/m^2^ was obtained off Shikoku (Fig. 3b), and another high heat flow of 97 ± 12 mW/m^2^ was obtained southwest of Kii Peninsula (Fig. 8c). A minimum of 25 ± 3 mW/m^2^ was observed off southern Hyuga (Fig. 3b). From offshore Tokai to Kii Peninsula at a distance of 15–25 km landward of the deformation front, the BSR-derived heat flow of this study ranged from 60 to 80 mW/m^2^ (Fig. 3b), and the heat flow from a probe ranged from 60 to 100 mW/m^2^ (Yamano et al. 2014). At a distance of 40–60 km landward of the deformation front, the BSR-derived heat flow of this study was 35–50 mW/m^2^, whereas the data from the probe measurements indicated heat flow values of 40–60 mW/m^2^ (Hamamoto et al. 2011). The differences between heat flow derived from our BSR and that from the probes used by Yamano et al. (2014) and Hamamoto et al. (2011) fell within the expected differences between BSR and probe values. We also confirmed that the differences between the heat flow values from the BSRs and those from the probes in neighboring areas fell within the range of 20%, except for two sites in the Kumano Basin where high heat flow values were observed (Fig. 8b). However, it should be noted that the number of probe measurements near the BSRs (within a distance of approximately 1 km) was limited, especially southwest of Kii Peninsula (Fig. 8a). The high heat flow from probe measurements at the Kumano Basin sites was thought to be affected by fluid expulsion (Hamamoto et al. 2011).

In convex-upward seafloor regions, the BSR was situated at a deeper depth; therefore, the heat flow values there were lower than in neighboring areas (Fig. 7). Rapid sedimentation causes low surface heat flow values, apparently due to a delay in recovery from the disturbed thermal equilibrium. However, sedimentation in the convex-upward seafloor regions was not likely to occur because of their geometry. Therefore, we considered that this low heat flow was caused by topography rather than rapid sedimentation. In contrast, in the convex-downward seafloor regions, the BSR was shallower and the values were higher than those in the surrounding areas (Fig. 7), apparently because rapid erosion caused high surface heat flow values. However, erosion in the convex-downward seafloor regions was not likely to occur because of their geometry. Therefore, we considered that this high heat flow was caused by topography rather than rapid erosion.

### Regional BSR depth variation

**Fig. 9:**
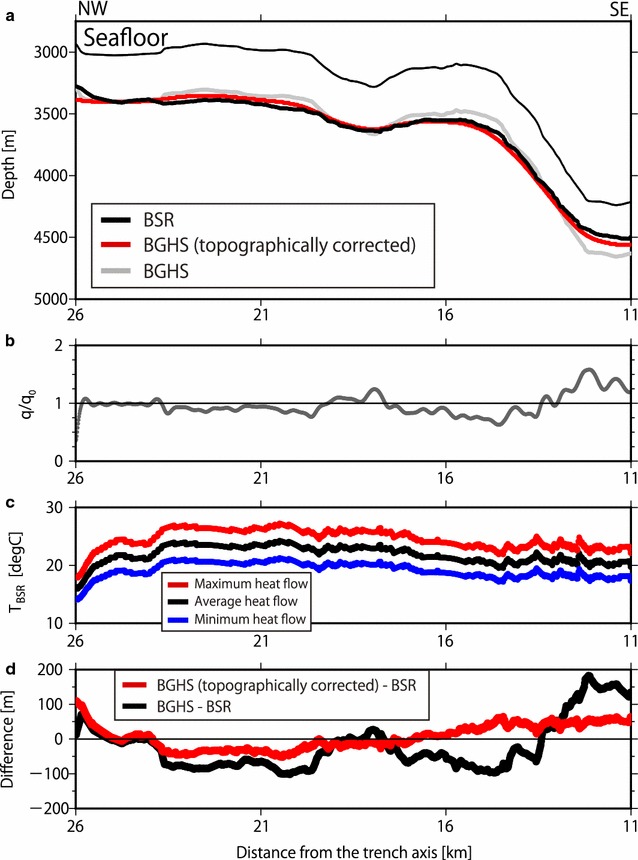
BSR and BGHS in the convex-upward and convex-downward seafloor regions southwest of the Kii Peninsula. **a** Bathymetry with the depths of the observed BSR and the calculated topographically corrected BGHS (2-D) and uncorrected BGHS (1-D). **b** Plot of $q/q_{0}$ ratio, where $q_{0}$ is the heat flow originating from the deep-seated heat flux, and $q$ is the heat flow estimated at the seafloor from the deep-seated heat flux. A ratio less than 1 indicates that the shallow subseafloor is subject to defocusing induced by the topography, and a ratio larger than 1 indicates that the shallow subseafloor is subject to focusing induced by the topography. **c** Error evaluation of the thermal modeling by comparison with the temperature of the BSR (*T*_BSR_) using maximum (red line), average (black line), and minimum (blue line) heat flow values. Temperature variations in the BSR fall within ± 3 °C. **d** Difference in depths between the observed BSR and calculated BGHS values with and without considering the topographic effect. The difference between the BSR and the two-dimensional BGHS values considering the topographic effect is smaller than that between the BSR and the one-dimensional BGHS without considering the effect

**Fig. 10:**
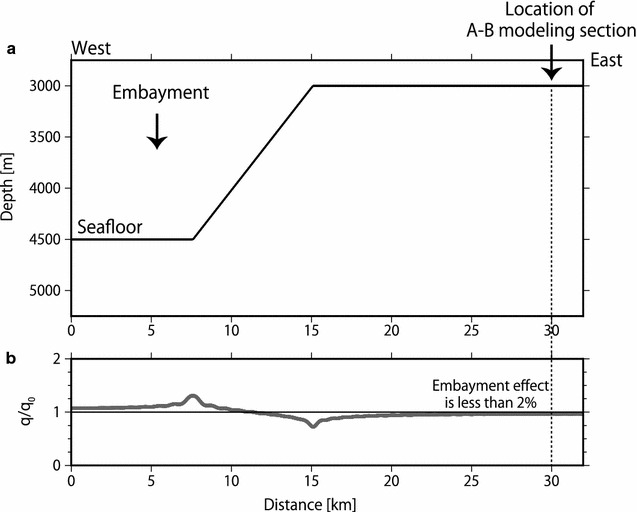
Effect of a large embayment southwest of the A–B section on surface heat flow. **a** Simplified bathymetry orthogonal to the A–B section. **b** Plot of $q/q_{0}$ ratio, where $q_{0}$ is the heat flow originating from the deep-seated heat flux, and $q$ is the estimated heat flow at the seafloor from the deep-seated heat flux. A ratio less than 1 indicates that the shallow subseafloor is subject to defocusing induced by the topography, whereas a ratio larger than 1 indicates that the shallow subseafloor is subject to focusing induced by the topography

The BSR depth variation in the convex-upward and convex-downward seafloor regions was investigated using two-dimensional thermal modeling in conjunction with topographic effects and seafloor temperature in the area where continuous BSRs were found. Twenty-one profiles were used to investigate the BSR depth variation in the study area (Fig. 9 and Additional file 1: Figures S4–S23). Based on this calculation, as expected, the convex-upward seafloor caused defocusing of the heat flow, whereas the convex-downward seafloor caused focusing of the heat flow (Fig. 9). This indicated that the BSRs were deeper in the convex-upward seafloor and shallower in the convex-downward seafloor than in the surrounding regions. A large embayment was present at the southwest of the modeling section A–B (Figs. 1, 8a), and this may have influenced surface heat flow of the A–B section. We evaluated the influence of the embayment on surface heat flow by evaluating only the section orthogonal to the A–B section. The result of this analysis indicated that the embayment (slope of 11° but far away from the modeling sections) influenced surface heat flow of the A–B section by less than 2%, assuming simplified bathymetry and constant deep-seated heat flux (Fig. 10). Here, we defined the BSR-derived heat flow by taking into account the influence of the undulating seafloor as the topographically corrected BSR-derived heat flow. The topographically corrected BSR-derived heat flow was obtained by dividing BSR-derived heat flow by the $q/q_{0}$ ratio, where $q_{0}$ and $q$ are, respectively, the heat flow originating from the deep-seated heat flux and the heat flow estimated at the seafloor from the deep-seated heat flux. After the topographic correction, the BSR-derived heat flow along the Kii Peninsula transect ranged from 30 to 80 mW/m^2^ at a distance of 10–50 km from the trench axis (Fig. 8c), while the uncorrected BSR-derived heat flow ranged from approximately 30–100 mW/m^2^. A sudden rise in the heat flow at a distance of 10–15 km from the trench axis was tempered with the topographic correction.

The estimated BGHS depths and observed BSR depths were expected to agree well. BGHS depths considering the topographic effect (hereafter referred to as “two-dimensional BGHS depths”) were defined as depths at the hydrate-gas phase boundary (Tishchenko et al. 2005) with increasing pressure and modeled temperature from the seafloor, whereas BGHS depths without considering topographic effect (hereafter referred to as “one-dimensional BGHS depths”) were depths at the phase boundary (Tishchenko et al. 2005) with linearly increasing pressure and temperature from the seafloor. As demonstrated in the previous section, BSR-derived heat flow values varied in the regions with a convex-upward or convex-downward seafloor. However, when considering the seafloor topography of the modeling sections, the influence appeared to propagate to only several hundreds of meters in the vertical direction and several kilometers in the horizontal direction. Therefore, the heat flow was predominantly determined by the basal heat flux from the subducting plate. We, therefore, conducted the thermal modeling by taking into account the topographic effect based on the heat flux. The two-dimensional BGHS depths calculated from this thermal modeling fit well with the observed BSR depths in both the convex-upward and convex-downward seafloor regions (Fig. 9d). Although the absolute difference between one-dimensional BGHS depth and the observed BSR depth reached 180 m at a distance of 11–16 km from the trench axis (Fig. 9d), the absolute difference between the two-dimensional BGHS depth and the BSR depth fell within 50 m in this region. Considering the uncertainty in the BSR depth (± 5%), the difference of 50 m was extended to approximately 30–70 m in this region. Similar results could also be found in other investigated profiles (Additional file 1: Figures S4–S23). These results indicated that the topographic effect should be one of the fundamental considerations in heat flow estimations and that our thermal modeling accurately expressed the local heat flow at least at depths between the seafloor and the BSR.

As demonstrated in the section discussing the uncertainties associated with this study, the total uncertainty in the heat flow from the BSRs was less than 25%. We also conducted an error evaluation of the thermal structure by comparing the maximum (+ 12.5%), average, and minimum (− 12.5%) basal heat flow (or temperature gradient, $\alpha$) values (Fig. 9c). The uncertainty yielded temperature variations on the BSR within ± 3 °C. These temperature variations also yielded variations in two-dimensional BGHS depths falling within ± 70 m.

## Discussion

### BSRs at the prism toe and trough floor off Tokai

We interpreted acoustic reflectors that parallel the seafloor with seismic reflection profiles showing high-amplitude reverse-polarity waveforms at the prism toe and at the trough (trench) floor as BSRs. To confirm whether these reflectors originated in methane hydrate, we calculated the theoretical depth of hydrate formation. The available heat flow values near these areas and those acquired at the trough floor with a thermal probe ranged from 80 to 100 mW/m^2^ (Yamano et al. 2003; Hamamoto et al. 2011). Assuming a thermal conductivity of 1.0 W/m/K, the one-dimensional BGHS depths were calculated to be 220–270 mbsf, while the observed BSR depth was 300–400 mbsf. Considering the absence of surface heat flow and P-wave velocity in the sediment very close to these areas (within distances of approximately 1 km) where BSRs were found, these reflectors could be interpreted to be methane hydrate BSRs. Artificial noises paralleling the seafloor, such as a waveform induced by airgun bubbles, were not observed throughout the region. Multiple reflectors could also be dismissed because of the discrepancy between the two depths.

### Heat flow variation

The heat flow was highly variable at a distance from the trench axis along the Kii Transect (Fig. 8c). The high BSR-derived heat flow value of 97 ± 12 mW/m^2^ was locally obtained along the lower landward slope in the southwestern region offshore of the Kii Peninsula, where the relatively young plate (Mahony et al. 2011) was subducting. This region is located in a convex-downward seafloor and is subject to the focusing effect induced by topography. The high heat flow value was estimated to be 61 ± 8 mW/m^2^ after topographic correction. The difference between the topographically uncorrected and corrected values was 36 ± 20 mW/m^2^ (Fig. 8c). This corrected heat flow value was not anomalously high compared with the surrounding areas. Therefore, the topographic effect was a primary contributor to the very small-scale variation in heat flow in this region.

The minimum BSR-derived heat flow of 25 ± 3 mW/m^2^ was obtained off southern Hyuga, where the oceanic plate was deeply subducting in the Nankai margin. This region is located in a convex-upward seafloor and is subject to the defocusing effects induced by topography. The minimum value of corrected heat flow was 29 ± 4 mW/m^2^. The slab deepening and the topography were likely to cause the minimum heat flow value in our study area. Although the sedimentation effect appeared to cause the lower heat flow (Hutchison 1985; Wang et al. 1993), according to our seismic profile, this region of low heat flow corresponded to ridge topography rather than a sedimentary basin.

### Possible causes of BSR depth anomalies other than topography

As mentioned in the previous section, the topographic effect was expected to be a primary contributor to the variation in heat flow values in convex-upward and convex-downward seafloor regions (Fig. 8c). After topographic correction, there were some sudden increases in BSR-derived heat flows throughout the modeling section, even far away from the lateral boundaries of the section (Fig. 8c, Additional file 1: Figure S24). These sudden increases may have reflected advective fluid flow where a thrust fault intersects the seafloor, although this fault cannot be confirmed from our seismic profiles. In order to obtain evidence for advective fluid flow, submersible exploration would be required. Therefore, the possibility of advection could not be excluded. Although recent erosion also causes sudden increases in heat flow, we could not confirm erosion from bathymetry features either. Moreover, sudden increases may be due to recent uplift, where thermal equilibrium had not yet been reached. Therefore, we may have actually overestimated heat flow values at some uplifted areas via topographic correction (e.g., at a distance of approximately 15 km from the trench axis in Additional file 1: Figure S14). More surveys that include measurements from probes and boreholes were needed to identify these phenomena.

The differences in BSR depths between our model and observations were large in some places. Although P-wave velocities that were different from the true velocity was one factor generating the differences, the influence was small because the BSR depths below the seafloor were shallow. For example, the error associated with BSR depths was 20 m assuming a P-wave velocity error of 100 m/s and a two-way travel time between the seafloor and the BSR of 0.4 s. We calculated the two-dimensional BGHS depths considering the 25% uncertainty demonstrated in the “Uncertainty in BSR-derived heat flow” section. In particular, we recalculated the thermal structure using the maximum (+ 12.5%) and minimum (− 12.5%) heat flows (geothermal gradient, $\alpha$) and calculated the two-dimensional BGHS depths from the recalculated thermal structure. We then calculated the corresponding differences between the recalculated two-dimensional BGHS and BSR. If we adopted the minimum absolute difference between the two-dimensional BGHS and BSR, most of the differences fell within 40 m (approximately one standard deviation of the differences between two-dimensional BGHS and BSR).

The differences that could not be explained by two-dimensional topographic effects, considering the uncertainty of BSR-derived heat flow, may have been due to prominent sedimentation and erosion. Sedimentation and erosion could be identified from the combination of geometries and positive and negative differences between the two-dimensional BGHS and BSR, although fluid flow, salinity variations, higher-order hydrocarbons, and three-dimensional topography could also explain the differences. If the calculated two-dimensional BGHS depths were shallower than the observed BSR depths, then the area was suggested to have suffered from the cooling effect of sedimentation. Likewise, calculated two-dimensional BGHS depths that were deeper than the observed depths suggested that the area suffered from erosion.

Sea level change also alters the depths of BSRs by inducing pressure changes in the water column (e.g., Kremer et al. 2017). However, a sea level change of 100 m produces only a 3.7% variation in heat flow at a water depth of 1500 m, assuming a uniform geothermal gradient of 40 °C/km (Expedition 315 Scientists 2009), if the BSR depths have not yet reached thermal equilibrium after the sea level change. Because the effect decreases with water depth and most of the modeling area was deeper than 1500 m, sea level change was negligible for estimations of sedimentation and erosion.

### Validity and significance of our thermal modeling

**Fig. 11:**
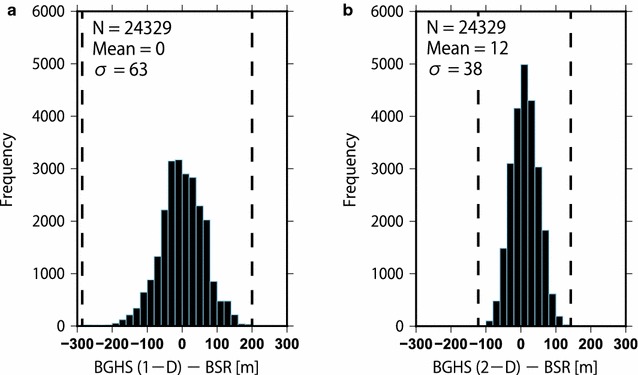
Differences between the BGHS and BSR depths, compiled from 21 seismic profiles (24,329 points). **a** Differences between the one-dimensional BGHS depths without considering the topographic effect and the BSR depths. The differences range from − 290 to + 200 m. The mean value and the standard deviation are 0 and 63 m, respectively. **b** Differences between the two-dimensional BGHS depths considering the topographic effect and the BSR depths. The differences range from − 110 to + 140 m. The mean value and the standard deviation are 12 and 38 m, respectively

We examined the differences between the two-dimensional BGHS depths and the observed BSR depths of all modeled sections in order to confirm whether the thermal modeling accurately expressed the subseafloor temperatures (Fig. 11). The differences between the two-dimensional BGHS depths from our thermal structure and the observed BSR depths ranged from − 110 to + 140 m (Fig. 11b), whereas, the differences in the one-dimensional BGHS depths (i.e., BGHS depths without considering the topographic effect) ranged from − 290 to + 200 m (Fig. 11a). This indicated that our modeled thermal structure that considers the topographic effect was more consistent with the subseafloor temperature estimated from the observed BSR depths.

Frictional heating along a subducting plate interface and radioactive heat production could change the thermal structure, even though the effective coefficient of friction was thought to be low in the Nankai Trough area (Yoshioka and Murakami 2007; Hamamoto et al. 2011; Ji et al. 2016). Most subduction zone thermal models do not consider topography with a wavelength of a few kilometers or less, as was treated by this study. In that case, the results of such a thermal model should be compared with heat flow observations that have been corrected for the small wavelength topography. The frictional heating and radioactive heat production could be estimated more precisely using a wide range of heat flow values in conjunction with our result for the shallow thermal structure because the topographic correction changed up to 36 mW/m^2^ of the BSR-derived heat flow in this study (Fig. 8c).

The time required to reach thermal equilibrium within subseafloor sequences through thermal diffusion is thought to be affected by the methane hydrate fraction and the latent heat of hydrate formation or dissociation (Kinoshita et al. 2011). Therefore, the sedimentation and erosion that occur within thermal equilibrium can be estimated by assuming the total hydrate fraction and an initial disturbance, such as rapid sedimentation or erosion. Although sedimentation and erosion can be estimated from anomalies in the local heat flow, erosion in convex-upward seafloor regions or sedimentation in convex-downward seafloor regions apparently mitigate local heat flow anomalies. In these areas, it is difficult to discern each phenomenon through only BSR depth data. However, comparing two-dimensional BGHS depths by considering the topographic effect in conjunction with the observed BSR depths can aid investigations of these phenomena. In summary, the shallow thermal structure that considers the topographic effect is of use in estimating the subseafloor temperature and also for understanding past surface geological phenomena, such as active sedimentation or erosion.

## Conclusions

We investigated the distributions of bottom-simulating reflectors (BSRs) in the landward region of the Nankai subduction zone. We also estimated heat flow from BSR depths, evaluated the error, and modeled the shallow thermal structure constrained by the temperature at BSR depths. The main results are summarized as follows.

1. BSRs were present across the area, ranging from the forearc basin to the accretionary prism slope. These were verified for the first time at the accretionary prism toe and at the trough floor offshore of the Tokai region.
2. BSR depths below the seafloor were larger in convex-upward seafloor regions and smaller in convex-downward seafloor regions than in neighboring areas. These regional variations could be explained by the defocusing and focusing effects induced by the topography. In addition, our model helps detect past surface geological phenomena, such as prominent sedimentation or erosion.
3. The total uncertainty of the BSR-derived heat flow should fall within 25%, considering allowable changes in the P-wave velocity in seawater and sediment, the geothermal gradient, and thermal resistance.

## Additional file

**Additional file 1.** Supporting Information regarding the location map of thermal modeling, heat flow profiles, and BSR-derived heat flow assuming lithostatic pressure. Thermal structure and BSR-derived heat flow in the Nankai subduction margin.

## Abbreviations

BSRs: bottom-simulating reflectors
BGHS: base of gas hydrate stability
MCS: multi-channel seismic
PHP: Philippine Sea plate
CMP: common mid-point
IODP: Integrated Ocean Drilling Program
ODP: Ocean Drilling Program
APCT3: advanced piston corer temperature tool
