# Supplementary material for: Distribution and depth of bottom-simulating reflectors in the Nankai subduction margin
Source: Earth Planets Space. 2018 Apr 17;70(1):60. doi: 10.1186/s40623-018-0833-5 (PMC5902540; doi:10.1186/s40623-018-0833-5)
Supplement: Supplementary file 1 — Additional file 1. Supporting Information regarding the location map of thermal modeling, heat flow profiles, and BSR-derived heat flow assuming lithostatic pressure. Thermal structure and BSR-derived heat flow in the Nankai subduction margin. [file 40623_2018_833_MOESM1_ESM.pdf]

Introduction

This supporting information provides 24 supporting figures. Figure S1 shows heat flow values calculated from BSRs assuming lithostatic pressure to compare with the results shown in main text assuming hydrostatic pressure. Figures S2 and S3 indicate the locations of estimated geothermal properties found in Figures S4–S23. Figures S4-S23 represent geothermal properties constrained by BSR depth temperature and seafloor topography in the Nankai subduction zone. Figure S24 represents heat flow profiles that show a sudden increase in topographically corrected BSR-derived heat flow.

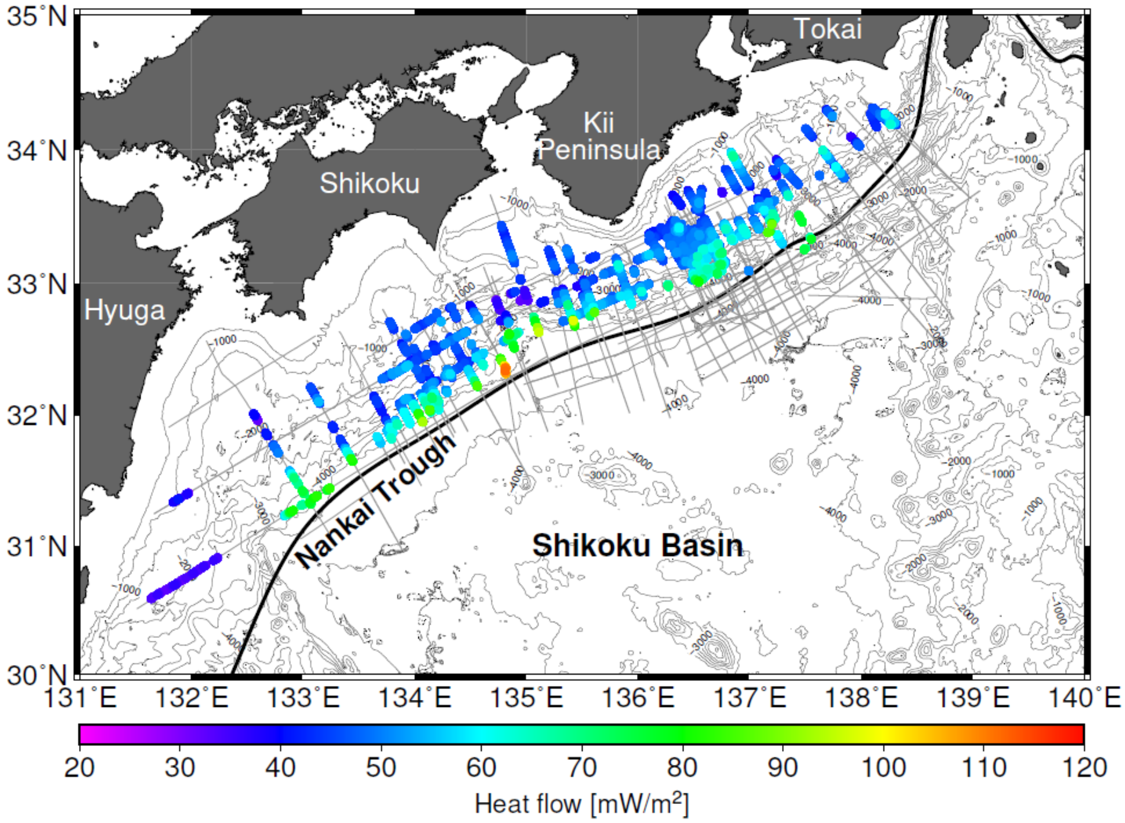

Figure S1. Map showing heat flow estimated from the BSRs assuming lithostatic pressure in the Nankai subduction zone. Gray lines represent MCS reflection surveys. Colored dots indicate BSR-derived heat flow.

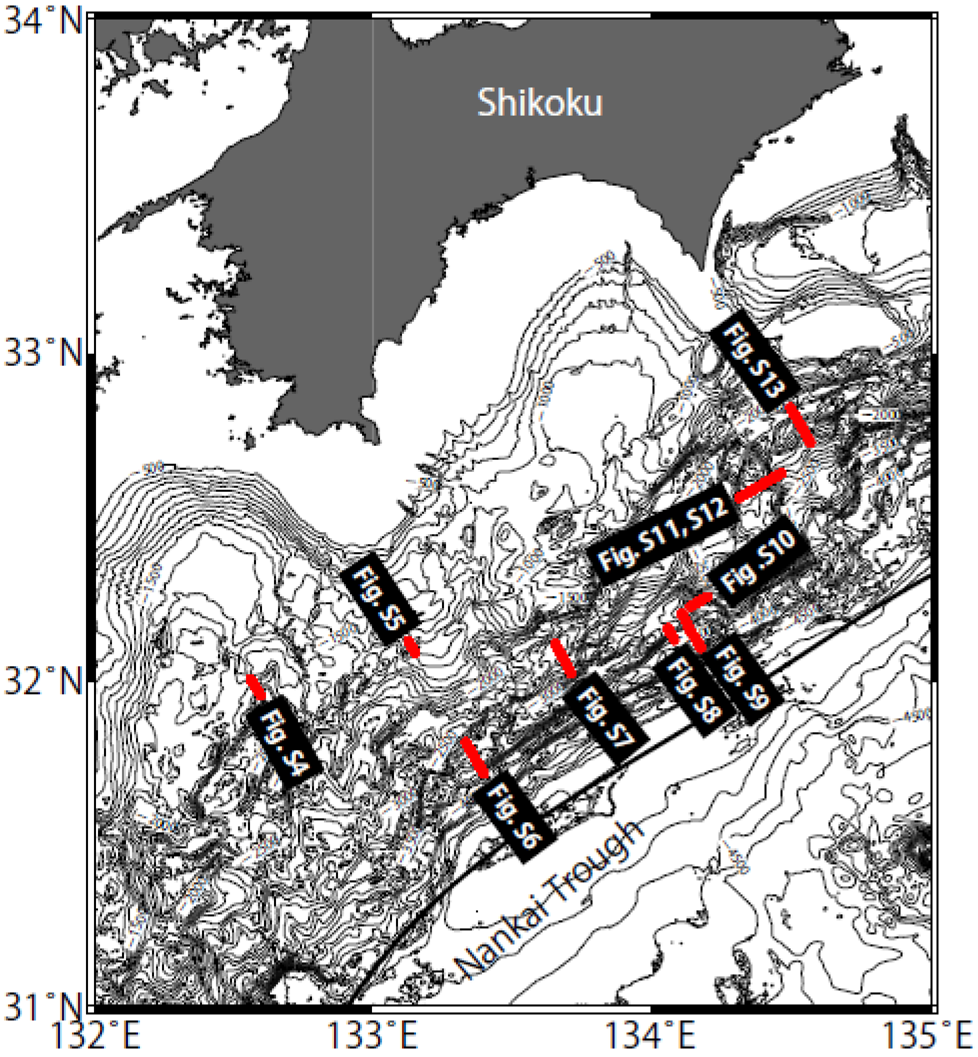

Figure S2. Map showing the locations of calculated thermal structures off Shikoku in the Nankai margin. Red lines indicate the sections of modeled thermal structures shown in Figures S4–S13.

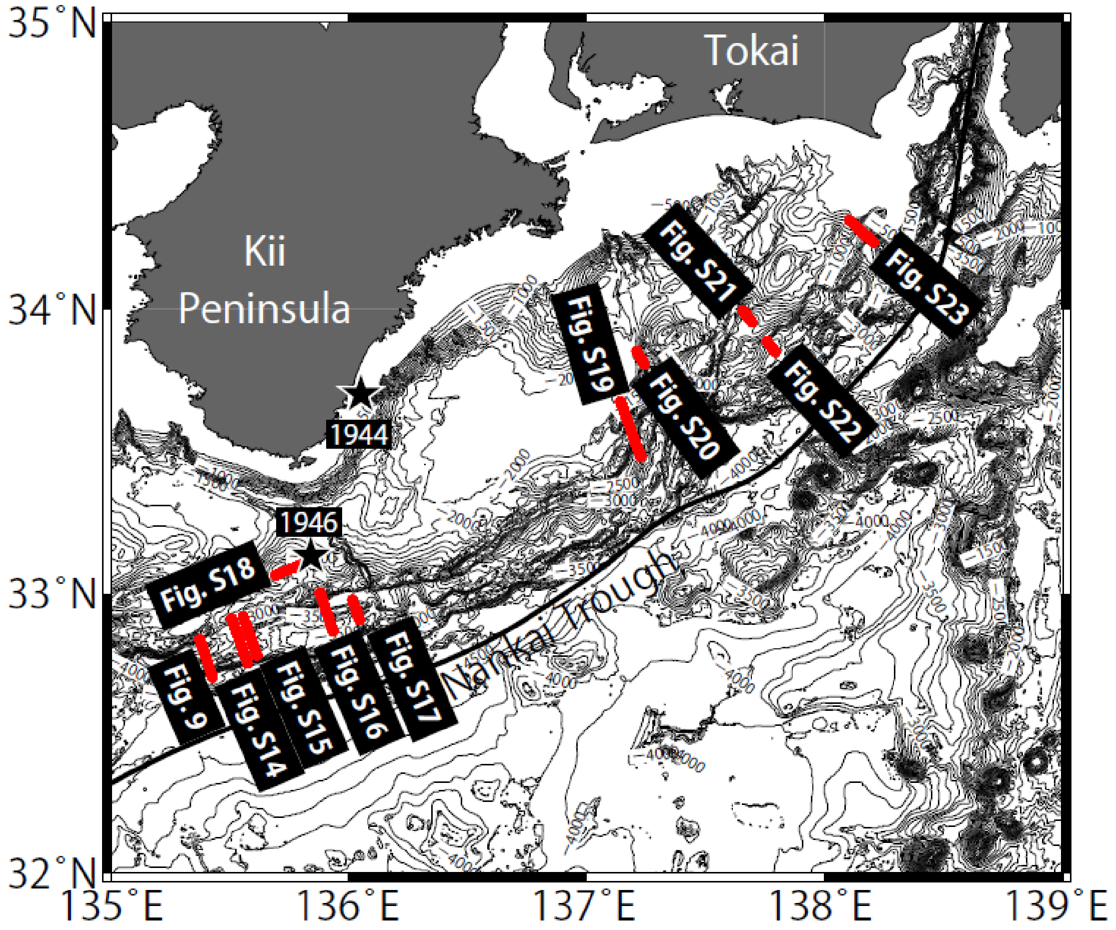

Figure S3. Map showing the locations of calculated thermal structures off Kii peninsula in the Nankai margin. Red lines indicate the sections of modeled thermal structures shown in Figures 9 and S14–S23. Black stars indicate the epicenters of the 1944 Tonankai earthquake and the 1946 Nankai earthquake [*Kanamori*, 1972].

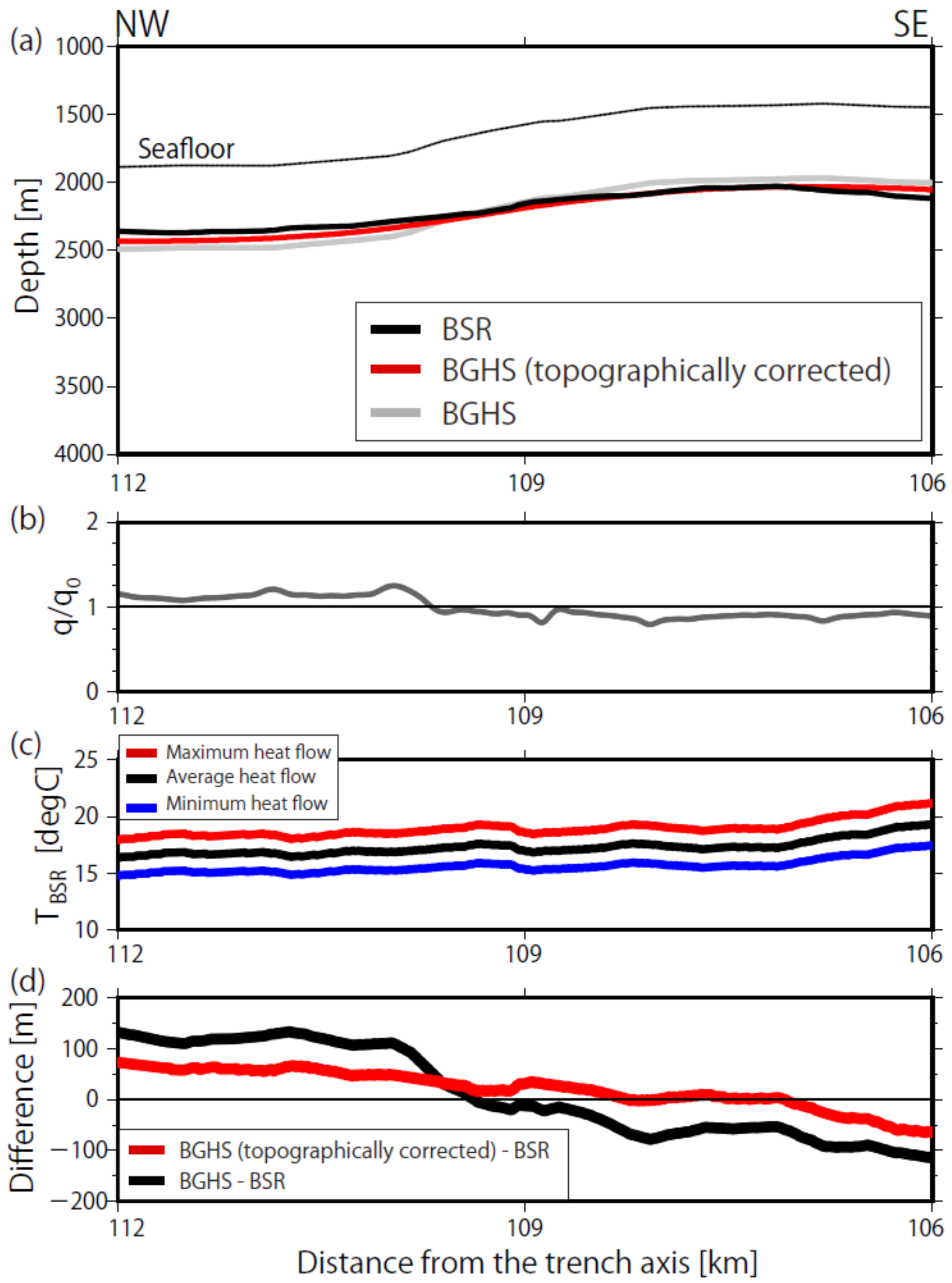

Figure S4. BSR and BGHS in the convex-upward and convex-downward seafloor regions at the location indicated as S4 in Figure S2. (a) Bathymetry with the depths of the observed BSR and the calculated topographically corrected (2-D) and uncorrected (1-D) BGHS. (b) A plot of q/ q_0 ratio, where q_0 is the heat flow originating from the deep-seated heat flux and q is estimated heat flow at the seafloor from deep-seated heat flux. (c) Error evaluation of the thermal modeling by comparison to temperature on the BSR (T_{BSR}) using maximum (red line), average (black line), and minimum (blue line) heat flow values. (d) Difference in depths between the observed BSR and calculated BGHS values with and without considering the topographic effect.

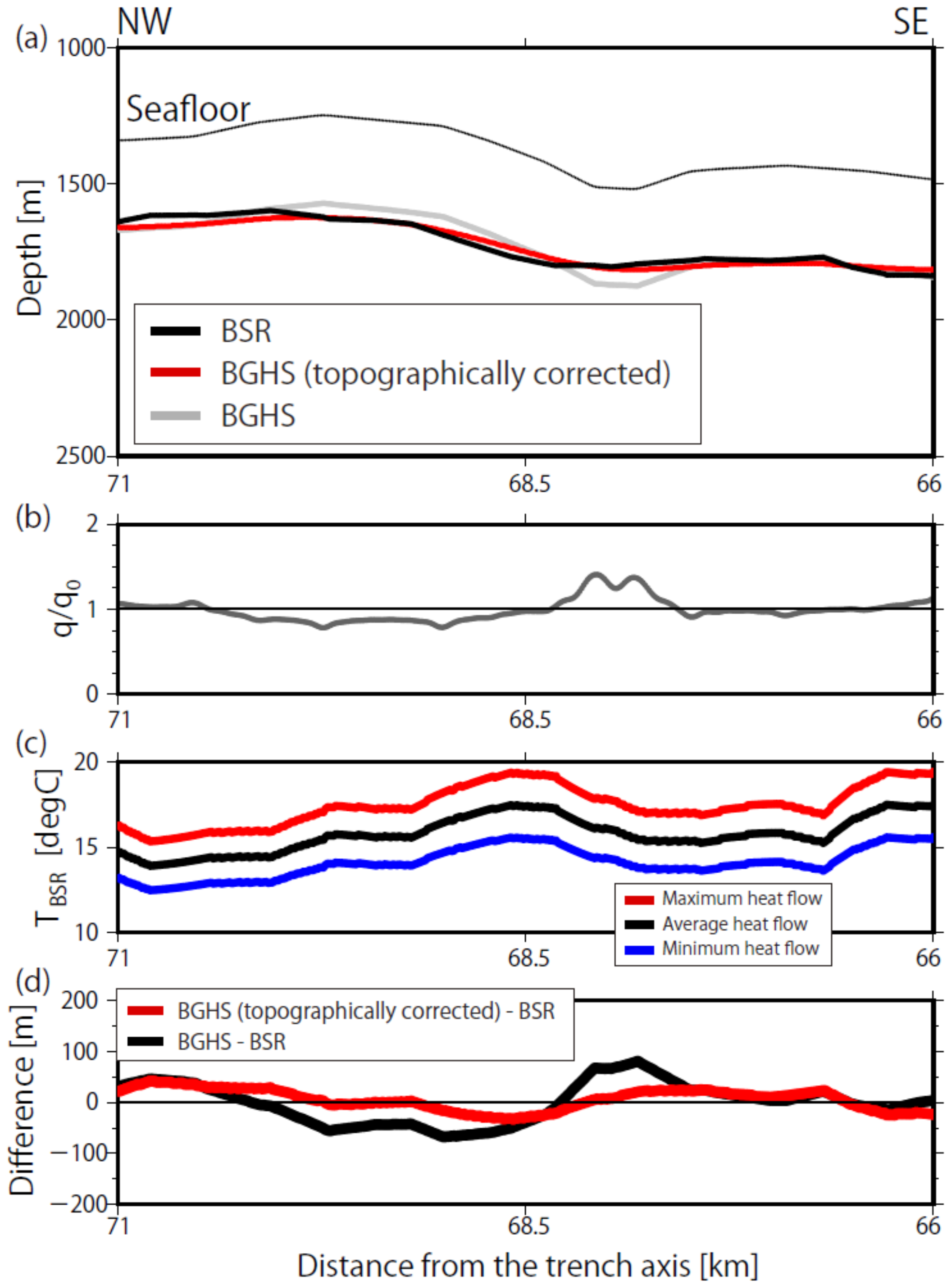

Figure S5. BSR and BGHS in the convex-upward and convex-downward seafloor regions at the location indicated as S5 in Figure S2. (a) Bathymetry with the depths of the observed BSR and the calculated topographically corrected (2-D) and uncorrected (1-D) BGHS. (b) A plot of $q/\ q_0$ ratio, where q_0 is the heat flow originating from the deep-seated heat flux and q is estimated heat flow at the seafloor from deep-seated heat flux. (c) Error evaluation of the thermal modeling by comparison to temperature on the BSR (T_{BSR}) using maximum (red line), average (black line), and minimum (blue line) heat flow values. (d) Difference in depths between the observed BSR and calculated BGHS values with and without considering the topographic effect.

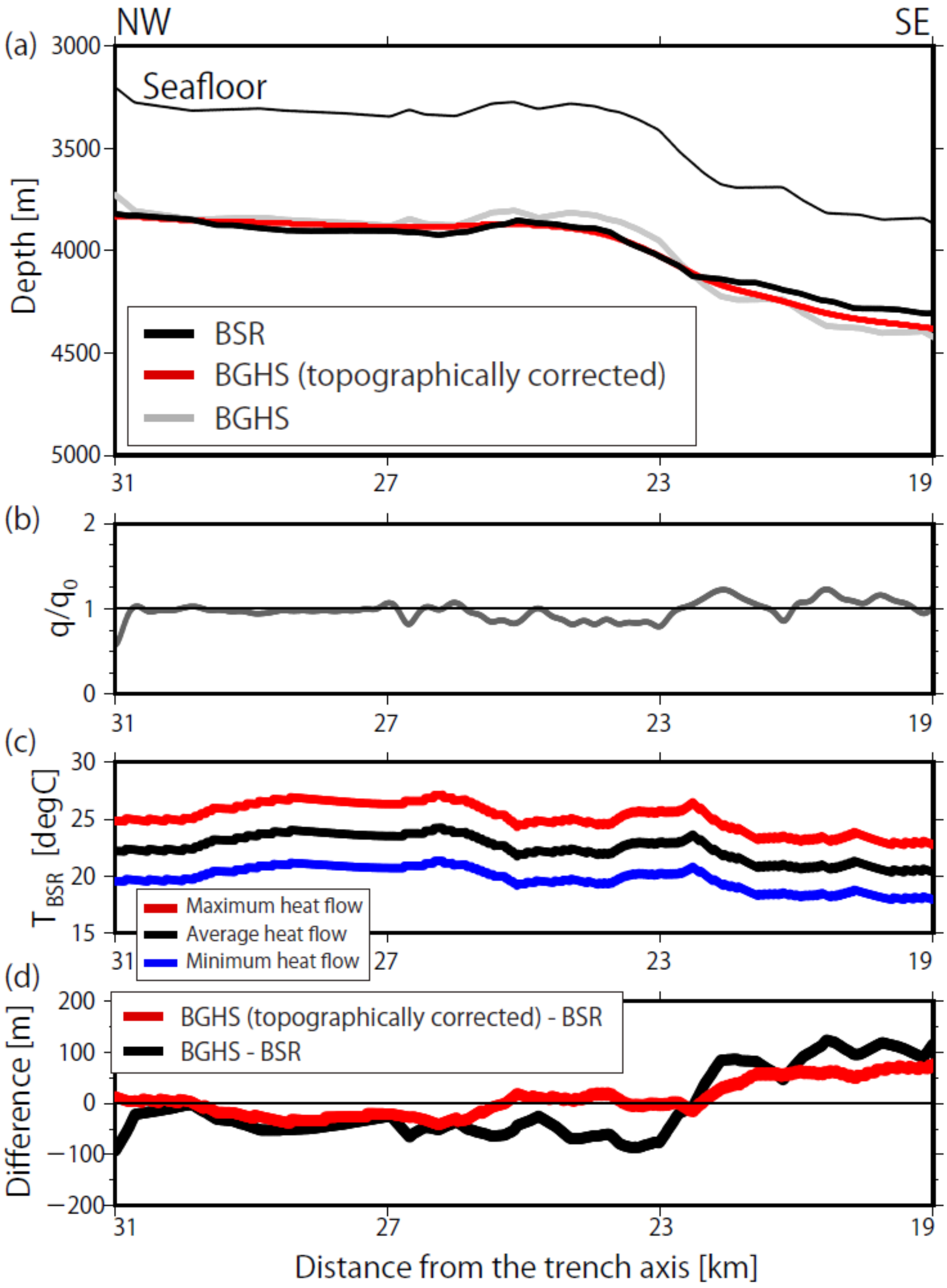

Figure S6. BSR and BGHS in the convex-upward and convex-downward seafloor regions at the location indicated as S6 in Figure S2. (a) Bathymetry with the depths of the observed BSR and the calculated topographically corrected (2-D) and uncorrected (1-D) BGHS. (b) A plot of q/ q_0 ratio, where q_0 is the heat flow originating from the deep-seated heat flux and q is estimated heat flow at the seafloor from deep-seated heat flux. (c) Error evaluation of the thermal modeling by comparison to temperature on the BSR (T_{BSR}) using maximum (red line), average (black line), and minimum (blue line) heat flow values. (d) Difference in depths between the observed BSR and calculated BGHS values with and without considering the topographic effect.

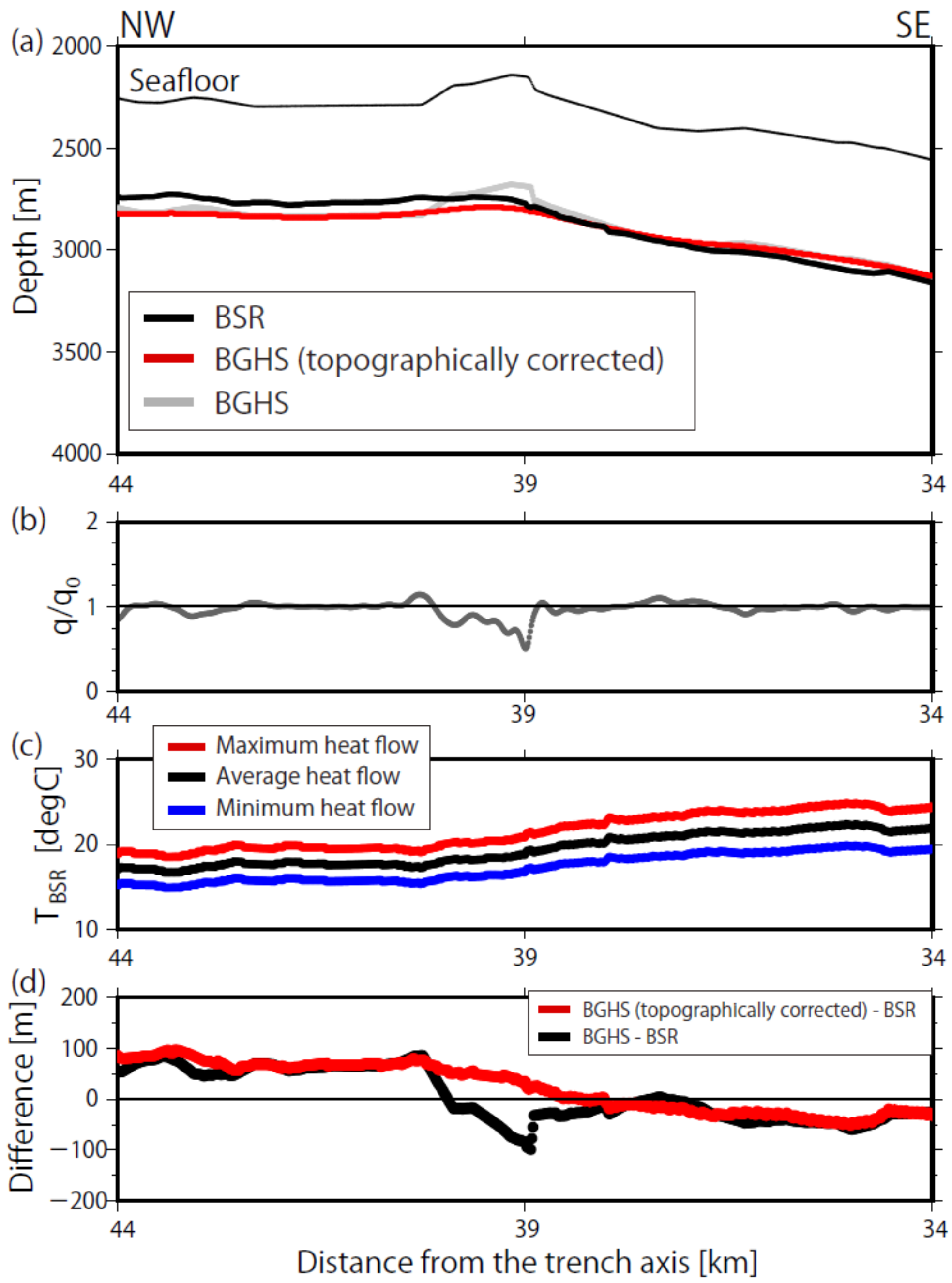

Figure S7. BSR and BGHS in the convex-upward and convex-downward seafloor regions at the location indicated as S7 in Figure S2. (a) Bathymetry with the depths of the observed BSR and the calculated topographically corrected (2-D) and uncorrected (1-D) BGHS. (b) A plot of q/ q_0 ratio, where q_0 is the heat flow originating from the deep-seated heat flux and q is estimated heat flow at the seafloor from deep-seated heat flux. (c) Error evaluation of the thermal modeling by comparison to temperature on the BSR (T_{BSR}) using maximum (red line), average (black line), and minimum (blue line) heat flow values. (d) Difference in depths between the observed BSR and calculated BGHS values with and without considering the topographic effect.

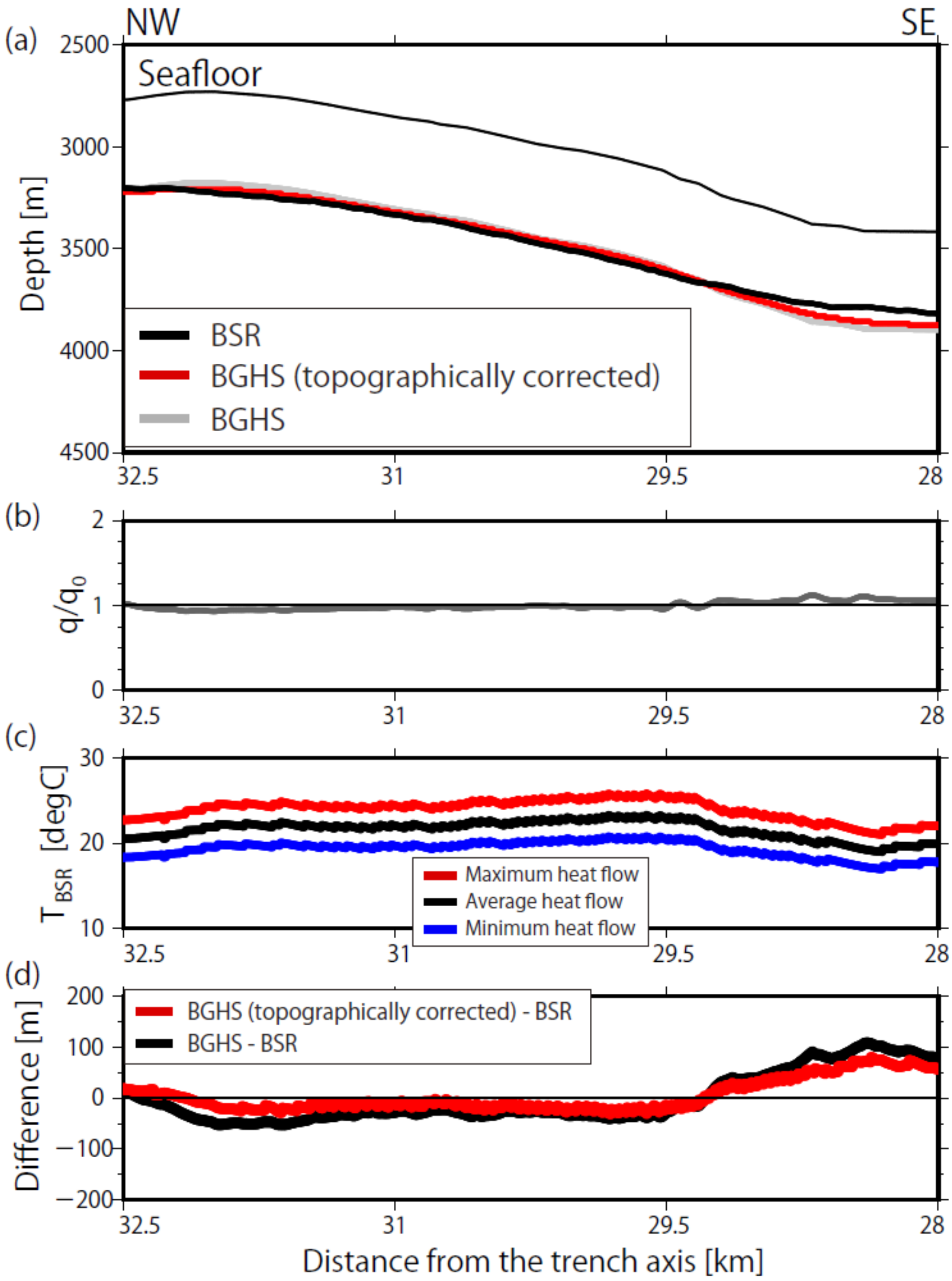

Figure S8. BSR and BGHS in the convex-upward and convex-downward seafloor regions at the location indicated as S8 in Figure S2. (a) Bathymetry with the depths of the observed BSR and the calculated topographically corrected (2-D) and uncorrected (1-D) BGHS. (b) A plot of q/q_0 ratio, where q_0 is the heat flow originating from the deep-seated heat flux and q is estimated heat flow at the seafloor from deep-seated heat flux. (c) Error evaluation of the thermal modeling by comparison to temperature on the BSR (T_{BSR}) using maximum (red line), average (black line), and minimum (blue line) heat flow values. (d) Difference in depths between the observed BSR and calculated BGHS values with and without considering the topographic effect.

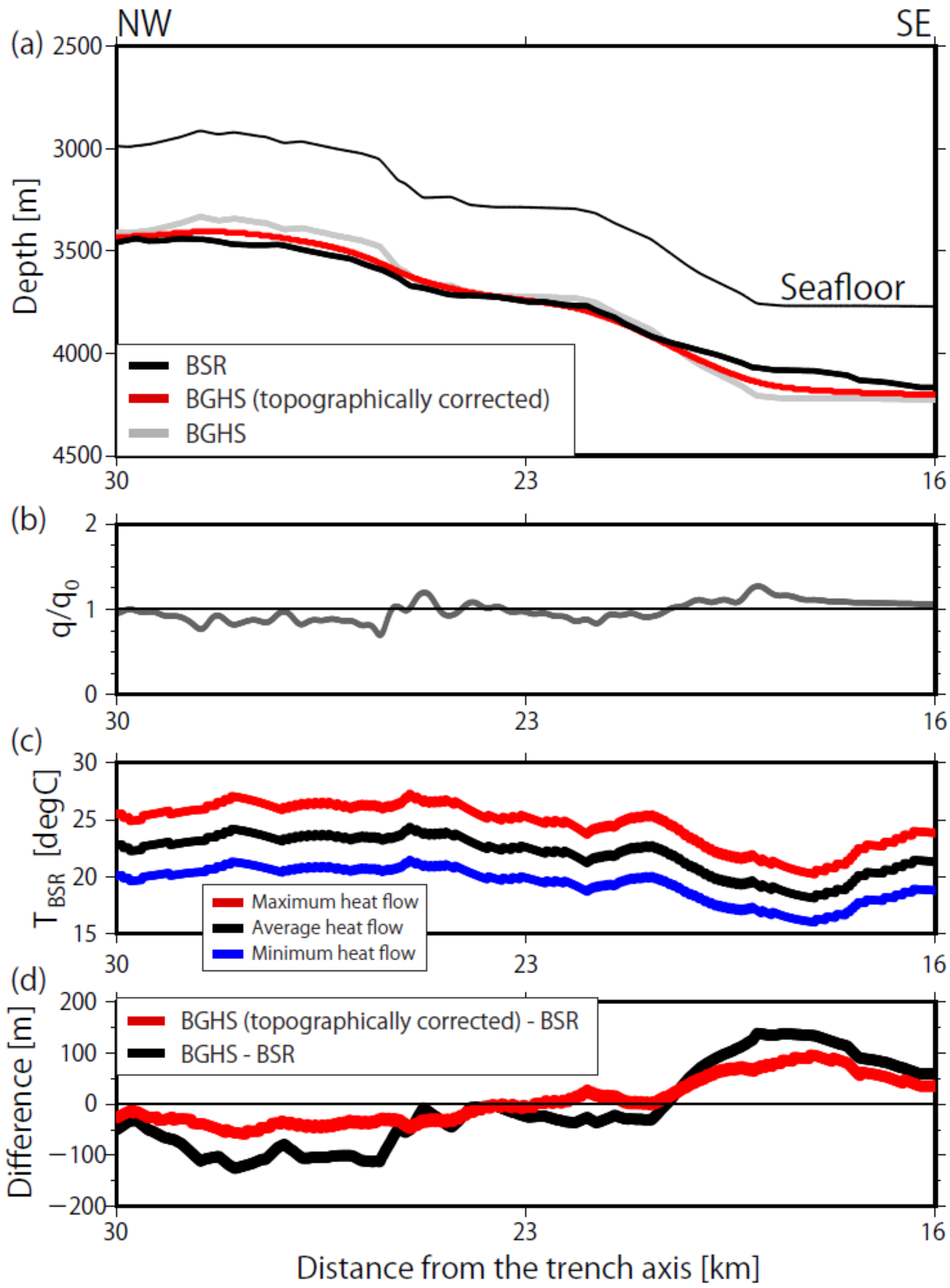

Figure S9. BSR and BGHS in the convex-upward and convex-downward seafloor regions at the location indicated as S9 in Figure S2. (a) Bathymetry with the depths of the observed BSR and the calculated topographically corrected (2-D) and uncorrected (1-D) BGHS. (b) A plot of q/q_0 ratio, where q_0 is the heat flow originating from the deep-seated heat flux and q is estimated heat flow at the seafloor from deep-seated heat flux. (c) Error evaluation of the thermal modeling by comparison to temperature on the BSR (T_{BSR}) using maximum (red line), average (black line), and minimum (blue line) heat flow values. (d) Difference in depths between the observed BSR and calculated BGHS values with and without considering the topographic effect.

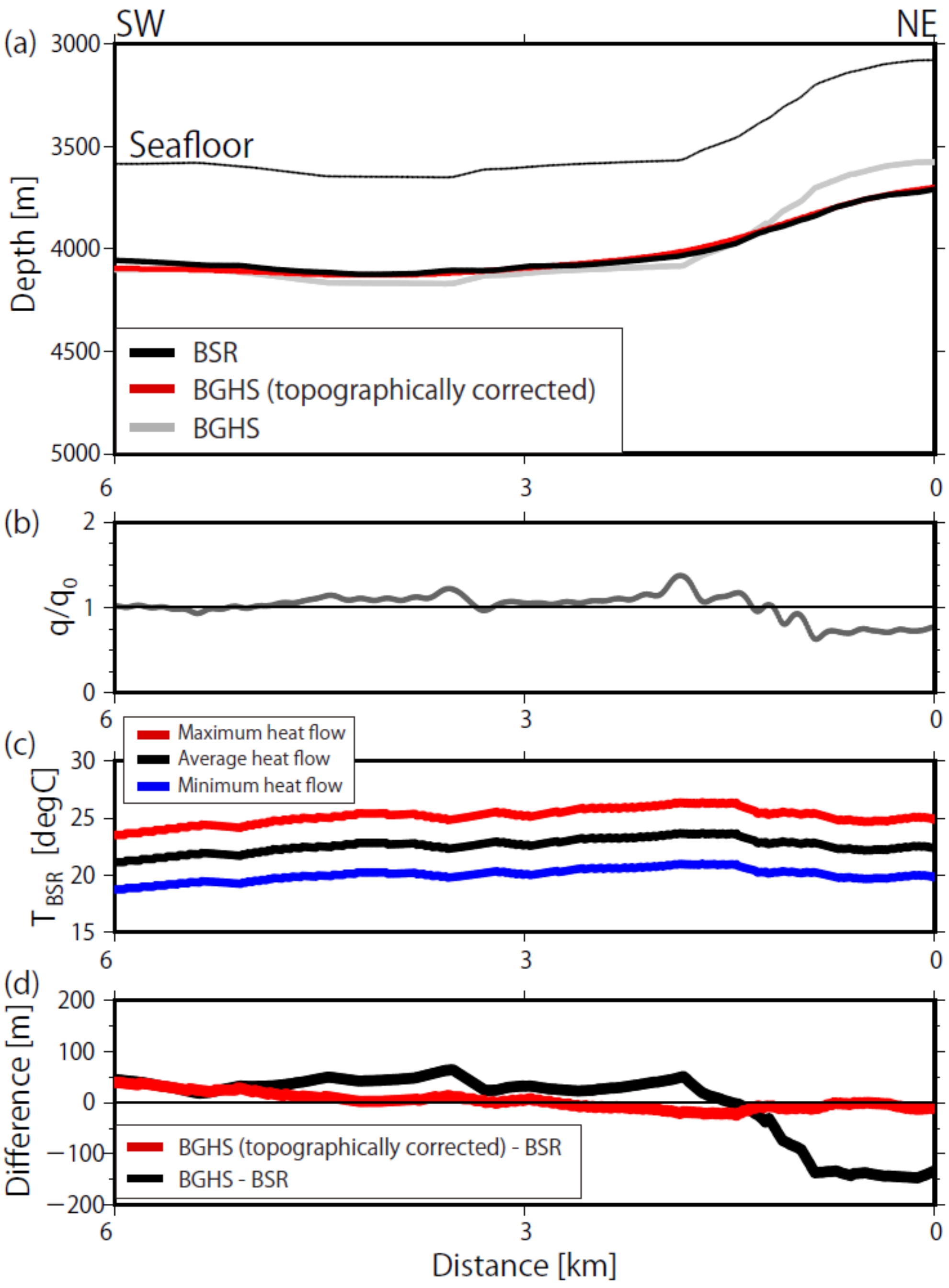

Figure S10. BSR and BGHS in the convex-upward and convex-downward seafloor regions at the location indicated as S10 in Figure S2. (a) Bathymetry with the depths of the observed BSR and the calculated topographically corrected (2-D) and uncorrected (1-D) BGHS. (b) A plot of q/ q_0 ratio, where q_0 is the heat flow originating from the deep-seated heat flux and q is estimated heat flow at the seafloor from deep-seated heat flux. (c) Error evaluation of the thermal modeling by comparison to temperature on the BSR (T_{BSR}) using maximum (red line), average (black line), and minimum (blue line) heat flow values. (d) Difference in depths between the observed BSR and calculated BGHS values with and without considering the topographic effect.

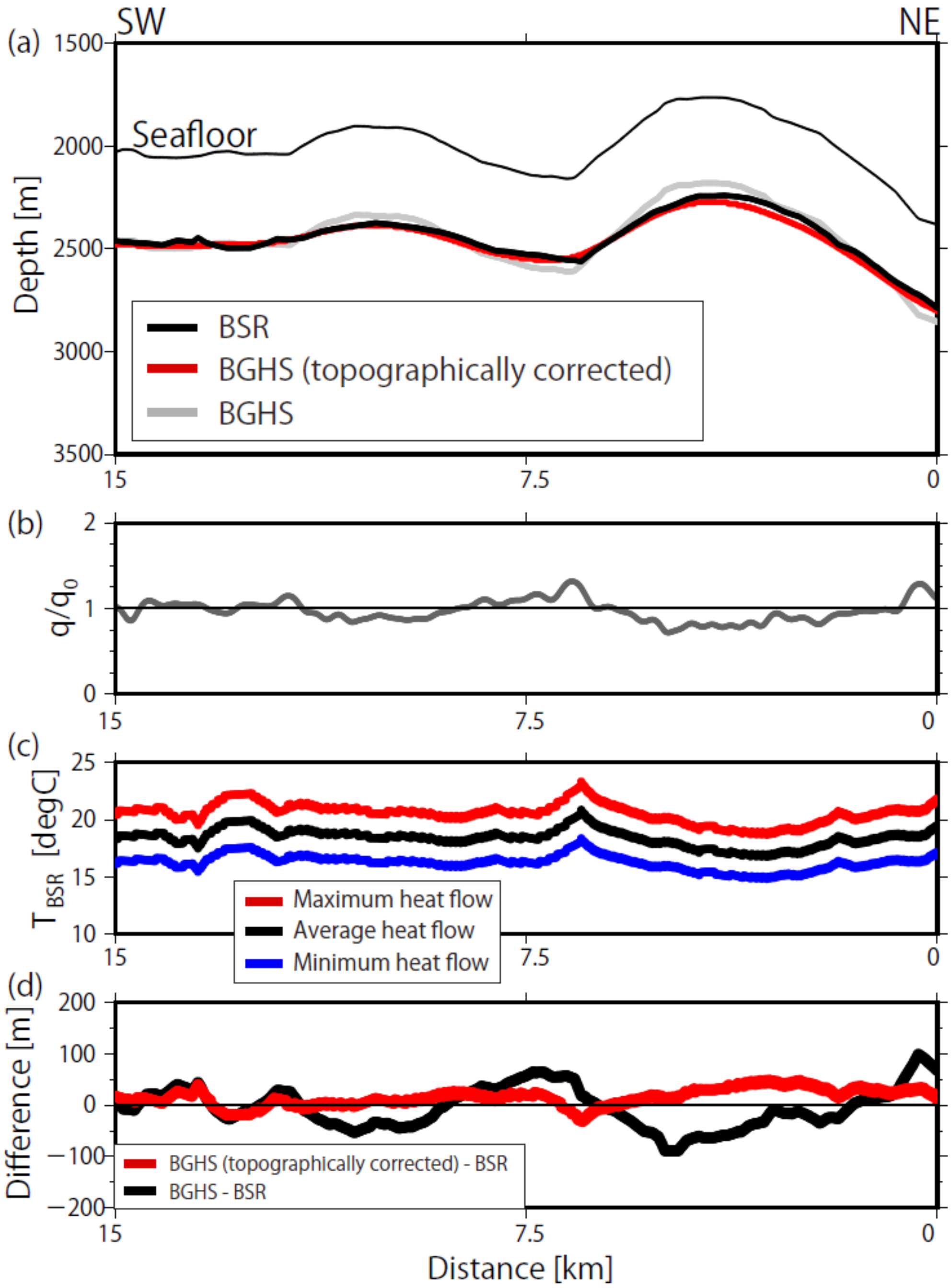

Figure S11. BSR and BGHS in the convex-upward and convex-downward seafloor regions at the location indicated as S11 in Figure S2. (a) Bathymetry with the depths of the observed BSR and the calculated topographically corrected (2-D) and uncorrected (1-D) BGHS. (b) A plot of q/ q_0 ratio, where q_0 is the heat flow originating from the deep-seated heat flux and q is estimated heat flow at the seafloor from deep-seated heat flux. (c) Error evaluation of the thermal modeling by comparison to temperature on the BSR (T_{BSR}) using maximum (red line), average (black line), and minimum (blue line) heat flow values. (d) Difference in depths between the observed BSR and calculated BGHS values with and without considering the topographic effect.

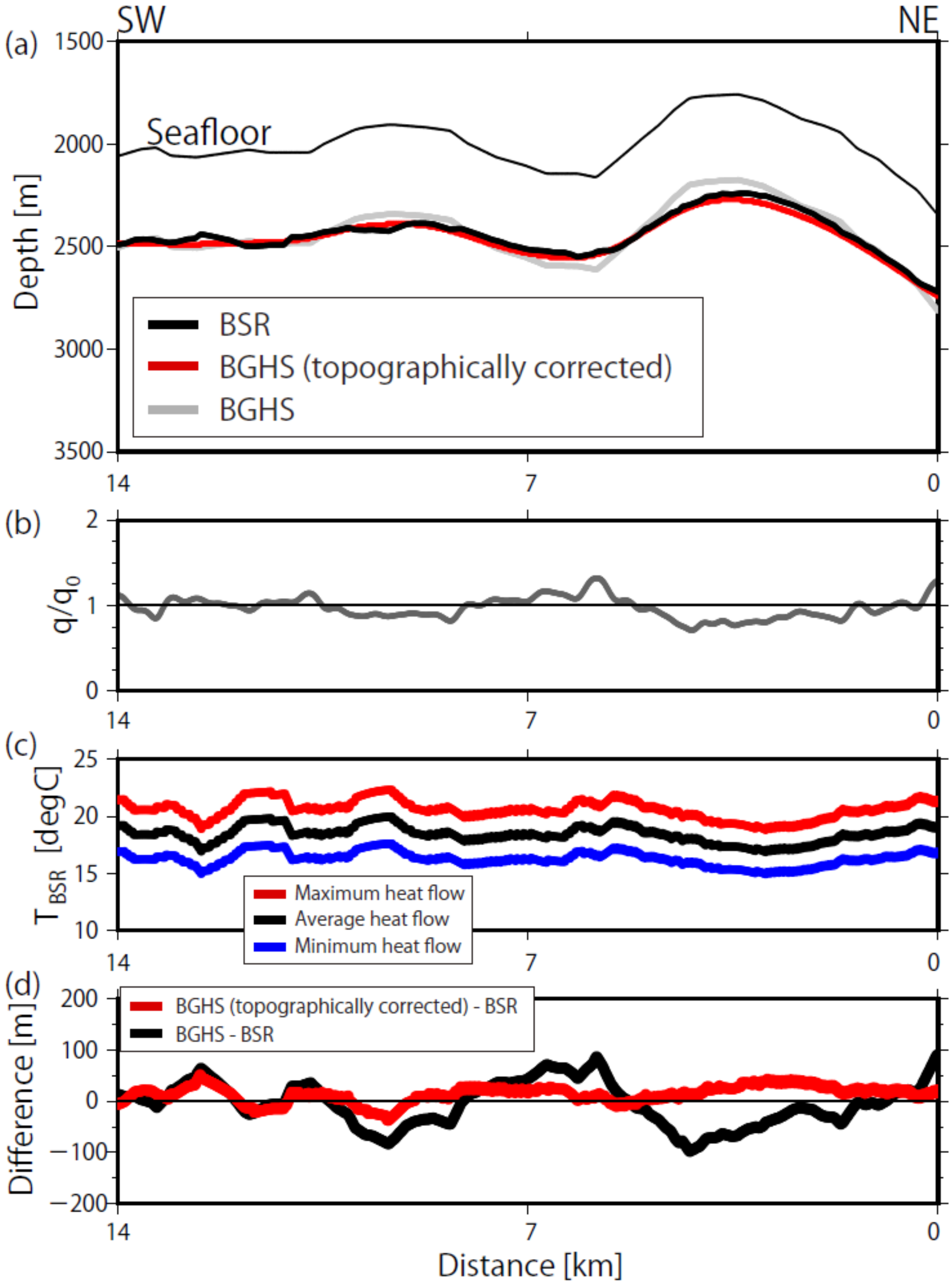

Figure S12. BSR and BGHS in the convex-upward and convex-downward seafloor regions at the location indicated as S12 in Figure S2. (a) Bathymetry with the depths of the observed BSR and the calculated topographically corrected (2-D) and uncorrected (1-D) BGHS. (b) A plot of q/ q_0 ratio, where q_0 is the heat flow originating from the deep-seated heat flux and q is estimated heat flow at the seafloor from deep-seated heat flux. (c) Error evaluation of the thermal modeling by comparison to temperature on the BSR (T_{BSR}) using maximum (red line), average (black line), and minimum (blue line) heat flow values. (d) Difference in depths between the observed BSR and calculated BGHS values with and without considering the topographic effect.

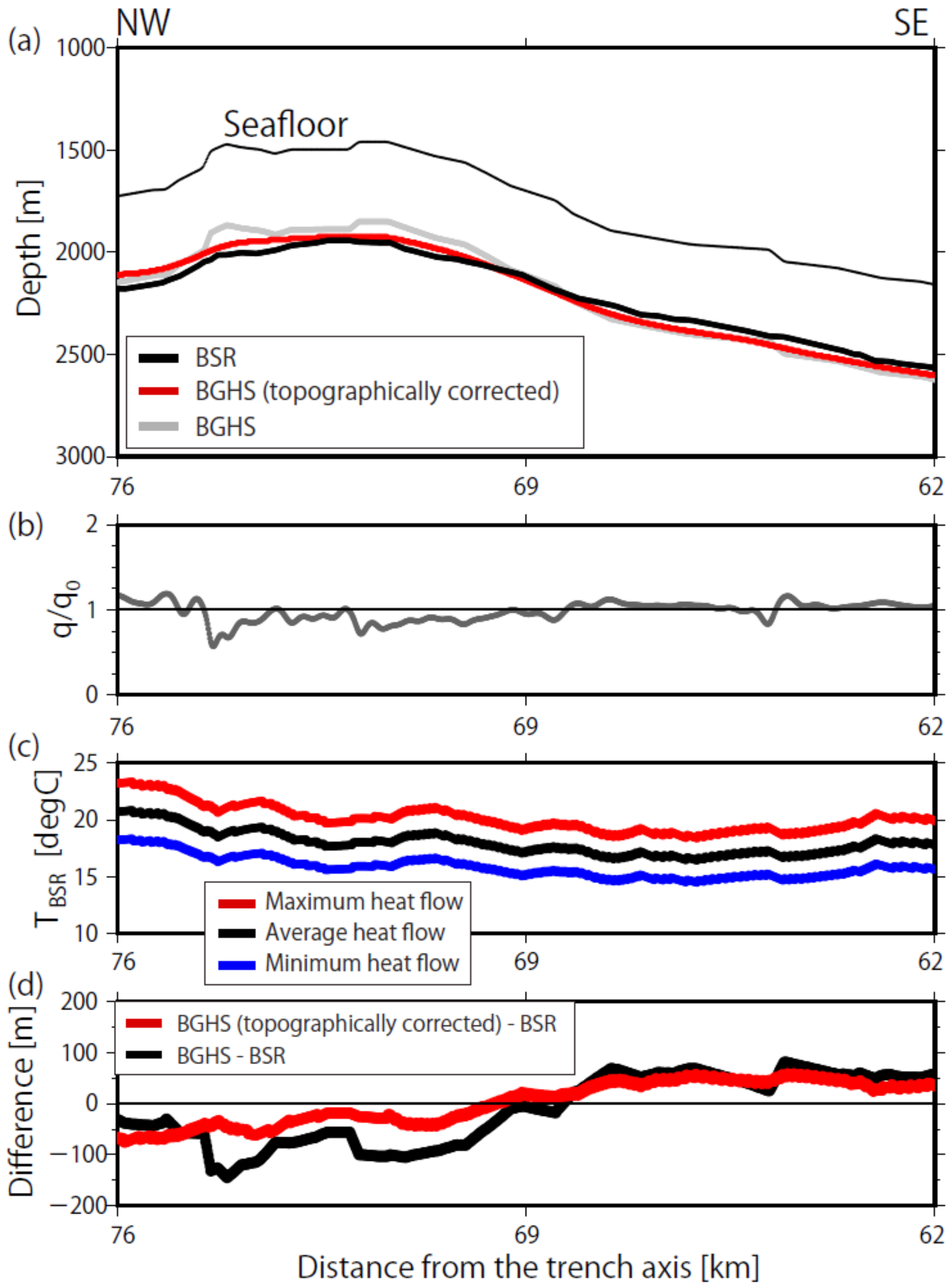

Figure S13. BSR and BGHS in the convex-upward and convex-downward seafloor regions at the location indicated as S13 in Figure S2. (a) Bathymetry with the depths of the observed BSR and the calculated topographically corrected (2-D) and uncorrected (1-D) BGHS. (b) A plot of q/q_0 ratio, where q_0 is the heat flow originating from the deep-seated heat flux and q is estimated heat flow at the seafloor from deep-seated heat flux. (c) Error evaluation of the thermal modeling by comparison to temperature on the BSR (T_{BSR}) using maximum (red line), average (black line), and minimum (blue line) heat flow values. (d) Difference in depths between the observed BSR and calculated BGHS values with and without considering the topographic effect.

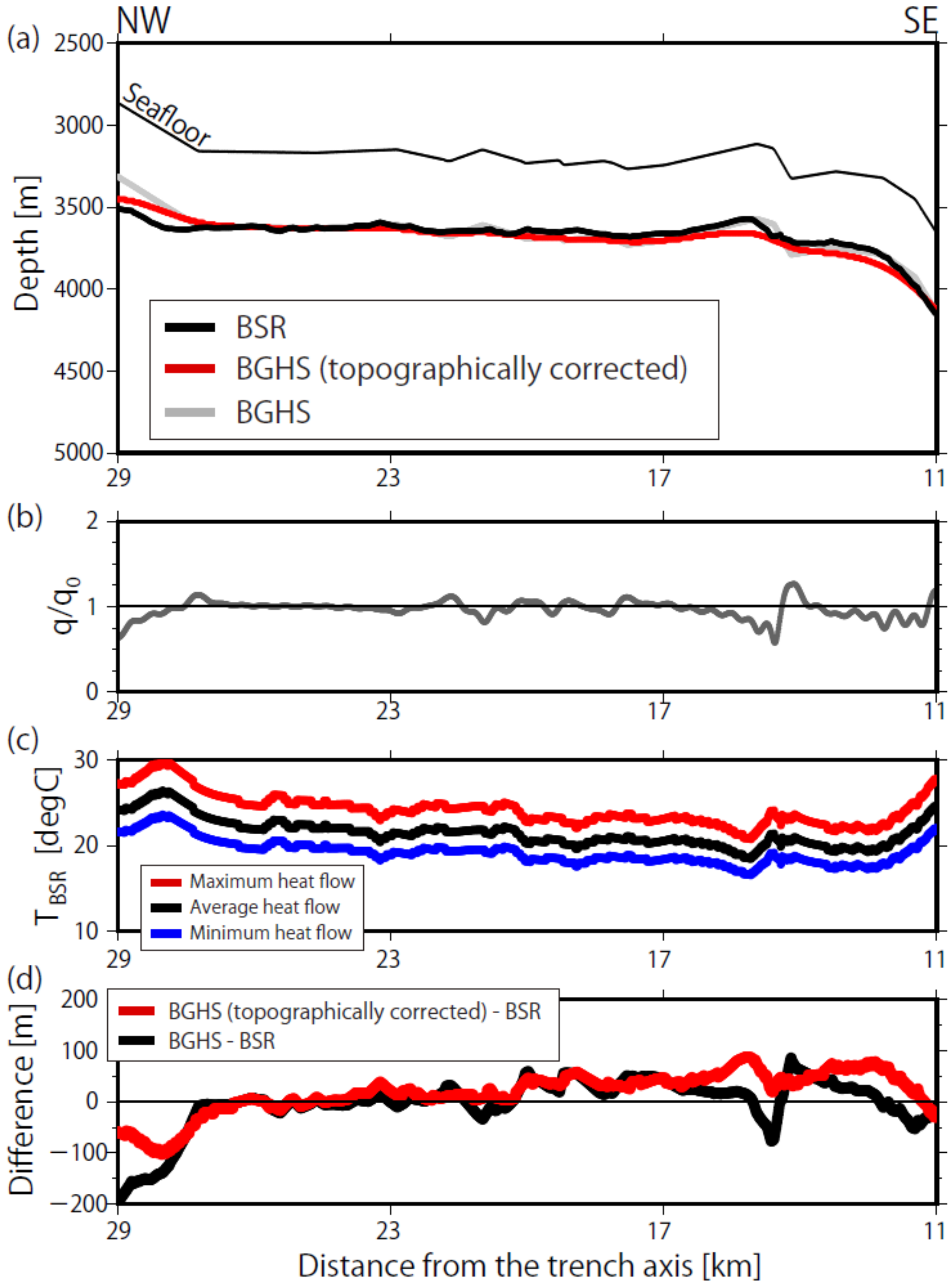

Figure S14. BSR and BGHS in the convex-upward and convex-downward seafloor regions at the location indicated as S14 in Figure S3. (a) Bathymetry with the depths of the observed BSR and the calculated topographically corrected (2-D) and uncorrected (1-D) BGHS. (b) A plot of q/ q_0 ratio, where q_0 is the heat flow originating from the deep-seated heat flux and q is estimated heat flow at the seafloor from deep-seated heat flux. (c) Error evaluation of the thermal modeling by comparison to temperature on the BSR (T_{BSR}) using maximum (red line), average (black line), and minimum (blue line) heat flow values. (d) Difference in depths between the observed BSR and calculated BGHS values with and without considering the topographic effect.

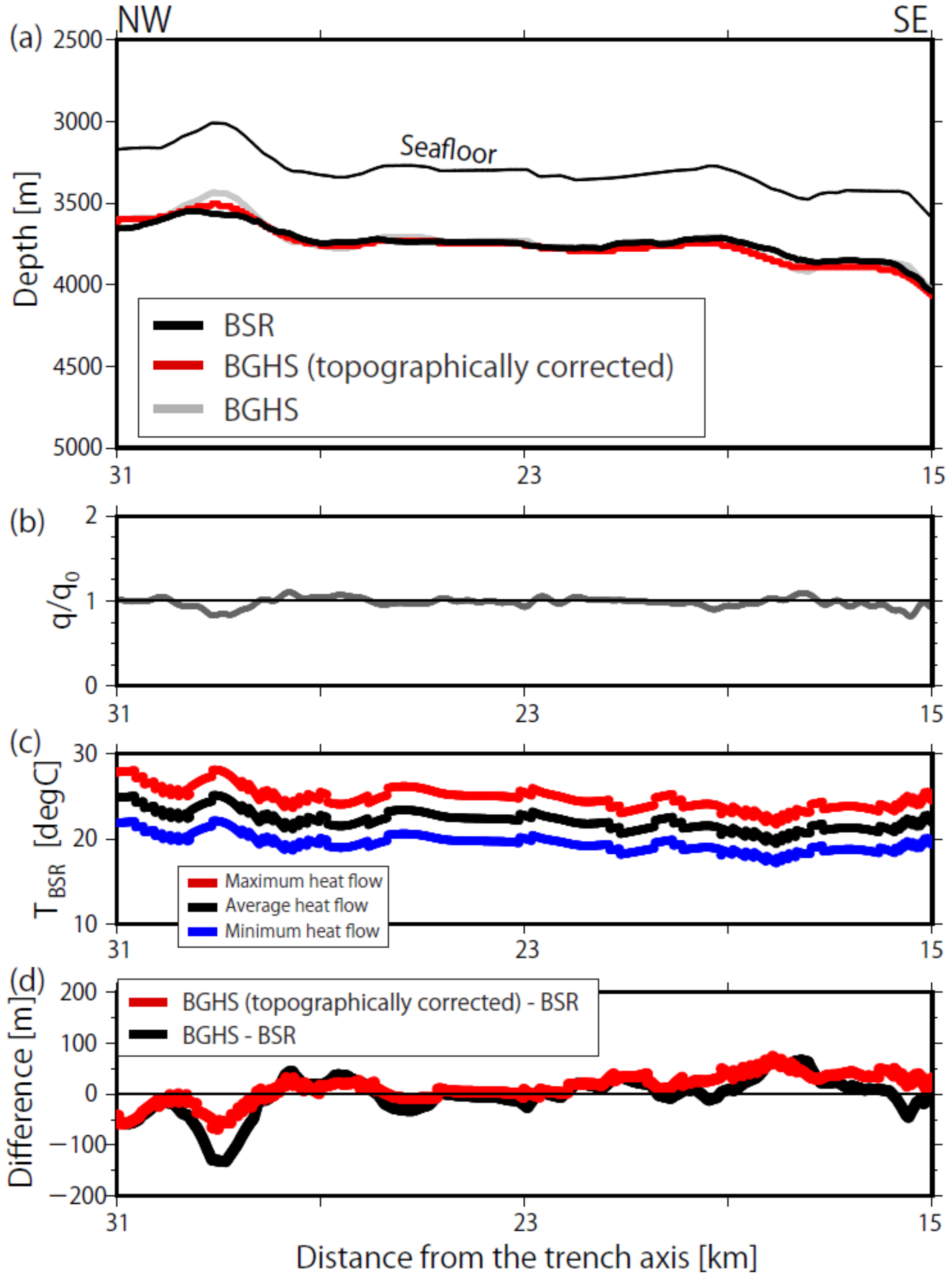

Figure S15. BSR and BGHS in the convex-upward and convex-downward seafloor regions at the location indicated as S15 in Figure S3. (a) Bathymetry with the depths of the observed BSR and the calculated topographically corrected (2-D) and uncorrected (1-D) BGHS. (b) A plot of q/ q_0 ratio, where q_0 is the heat flow originating from the deep-seated heat flux and q is estimated heat flow at the seafloor from deep-seated heat flux. (c) Error evaluation of the thermal modeling by comparison to temperature on the BSR (T_{BSR}) using maximum (red line), average (black line), and minimum (blue line) heat flow values. (d) Difference in depths between the observed BSR and calculated BGHS values with and without considering the topographic effect.

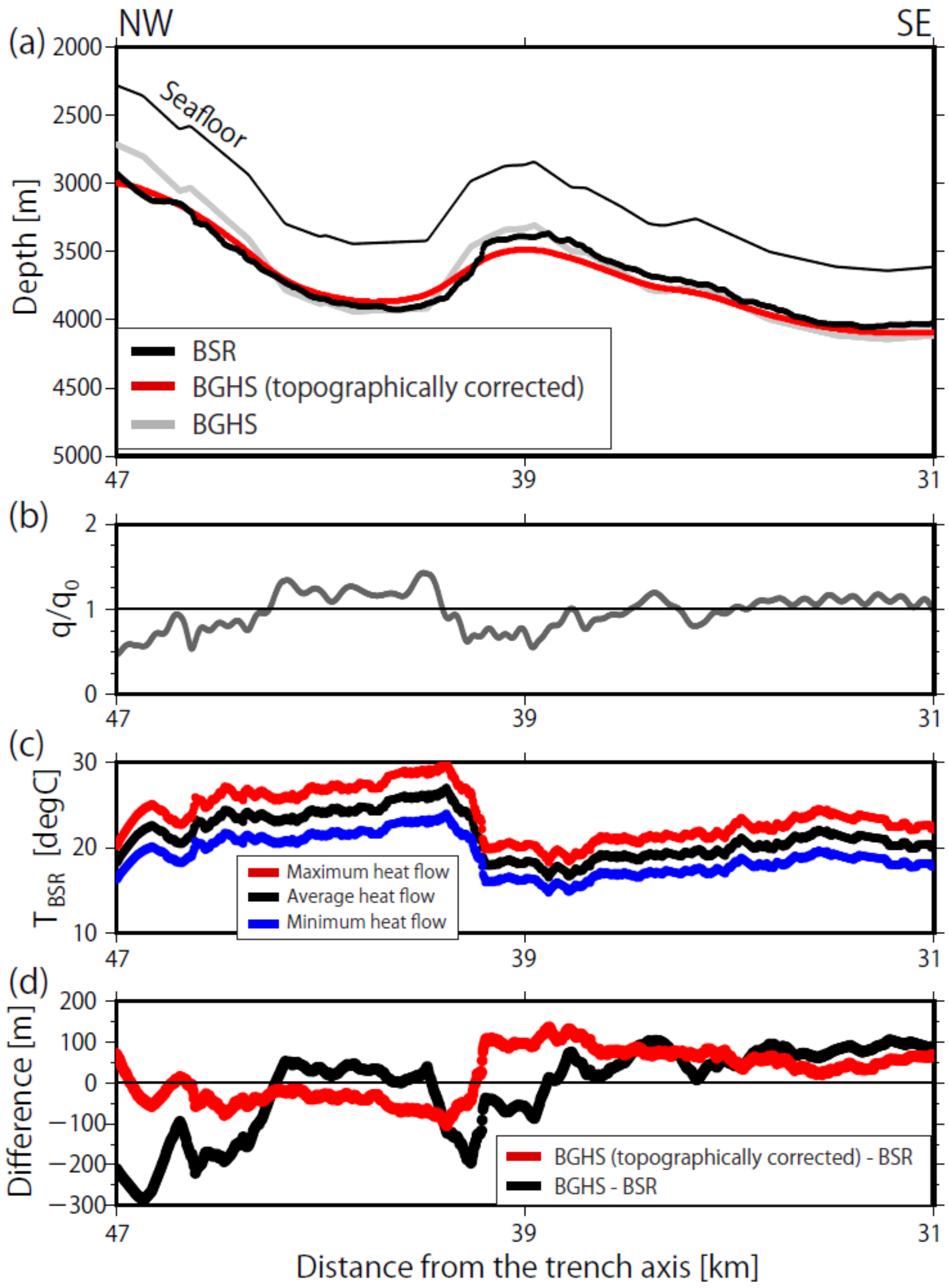

Figure S16. BSR and BGHS in the convex-upward and convex-downward seafloor regions at the location indicated as S16 in Figure S3. (a) Bathymetry with the depths of the observed BSR and the calculated topographically corrected (2-D) and uncorrected (1-D) BGHS. (b) A plot of q/ q_0 ratio, where q_0 is the heat flow originating from the deep-seated heat flux and q is estimated heat flow at the seafloor from deep-seated heat flux. (c) Error evaluation of the thermal modeling by comparison to temperature on the BSR (T_{BSR}) using maximum (red line), average (black line), and minimum (blue line) heat flow values. (d) Difference in depths between the observed BSR and calculated BGHS values with and without considering the topographic effect.

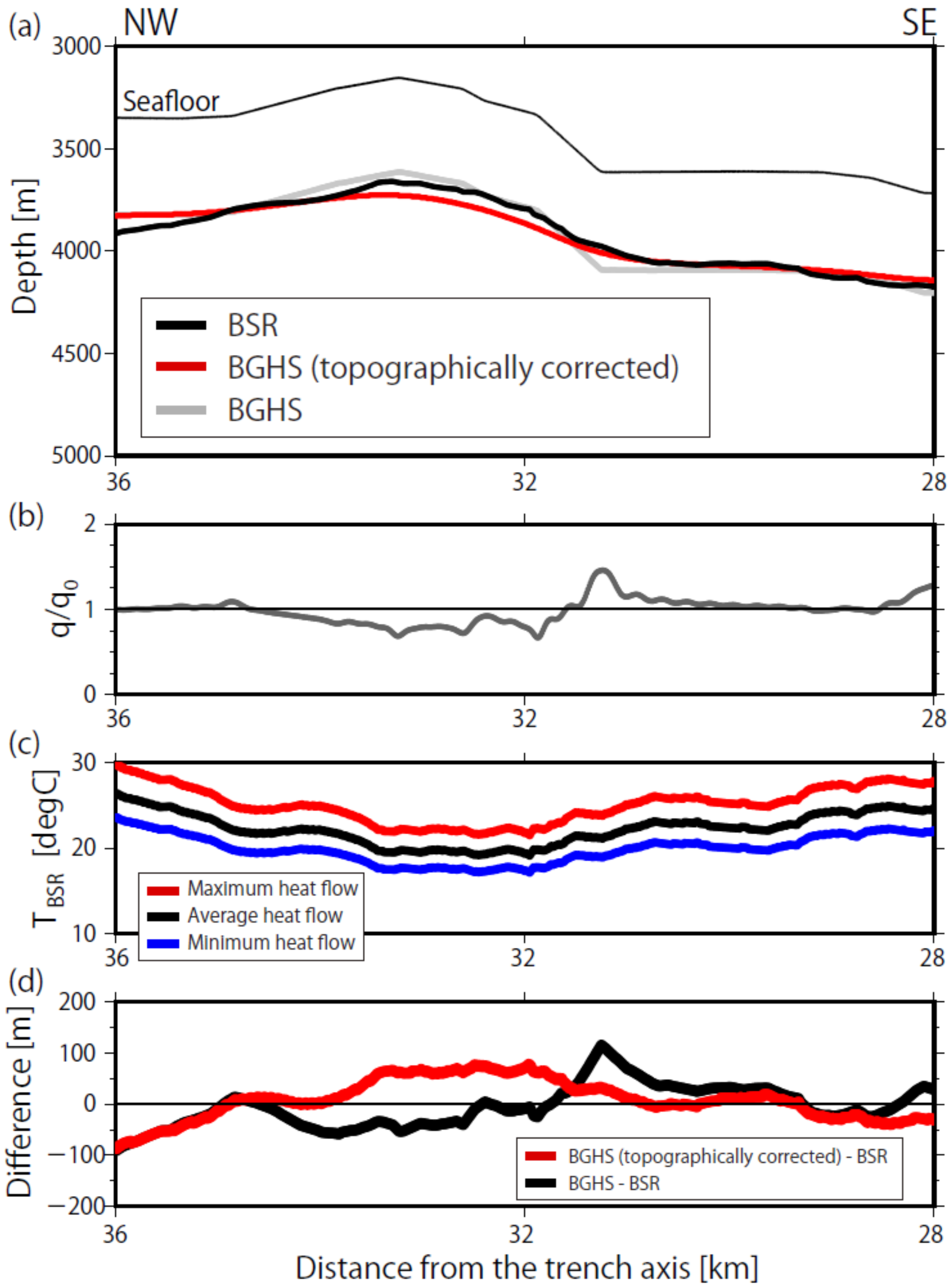

Figure S17. BSR and BGHS in the convex-upward and convex-downward seafloor regions at the location indicated as S17 in Figure S3. (a) Bathymetry with the depths of the observed BSR and the calculated topographically corrected (2-D) and uncorrected (1-D) BGHS. (b) A plot of q/ q_0 ratio, where q_0 is the heat flow originating from the deep-seated heat flux and q is estimated heat flow at the seafloor from deep-seated heat flux. (c) Error evaluation of the thermal modeling by comparison to temperature on the BSR (T_{BSR}) using maximum (red line), average (black line), and minimum (blue line) heat flow values. (d) Difference in depths between the observed BSR and calculated BGHS values with and without considering the topographic effect.

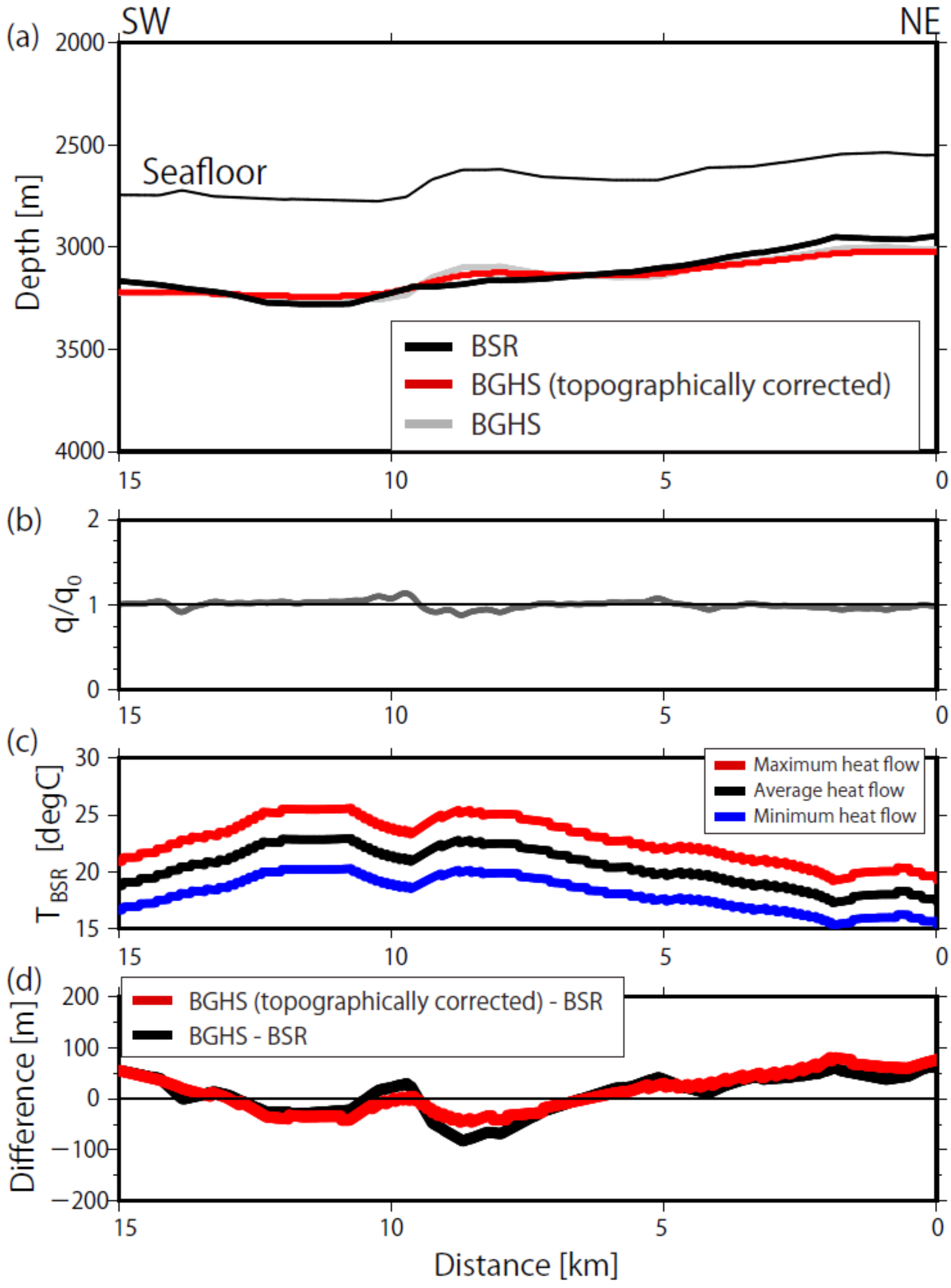

Figure S18. BSR and BGHS in the convex-upward and convex-downward seafloor regions at the location indicated as S18 in Figure S3. (a) Bathymetry with the depths of the observed BSR and the calculated topographically corrected (2-D) and uncorrected (1-D) BGHS. (b) A plot of q/q_0 ratio, where q_0 is the heat flow originating from the deep-seated heat flux and q is estimated heat flow at the seafloor from deep-seated heat flux. (c) Error evaluation of the thermal modeling by comparison to temperature on the BSR (T_{BSR}) using maximum (red line), average (black line), and minimum (blue line) heat flow values. (d) Difference in depths between the observed BSR and calculated BGHS values with and without considering the topographic effect.

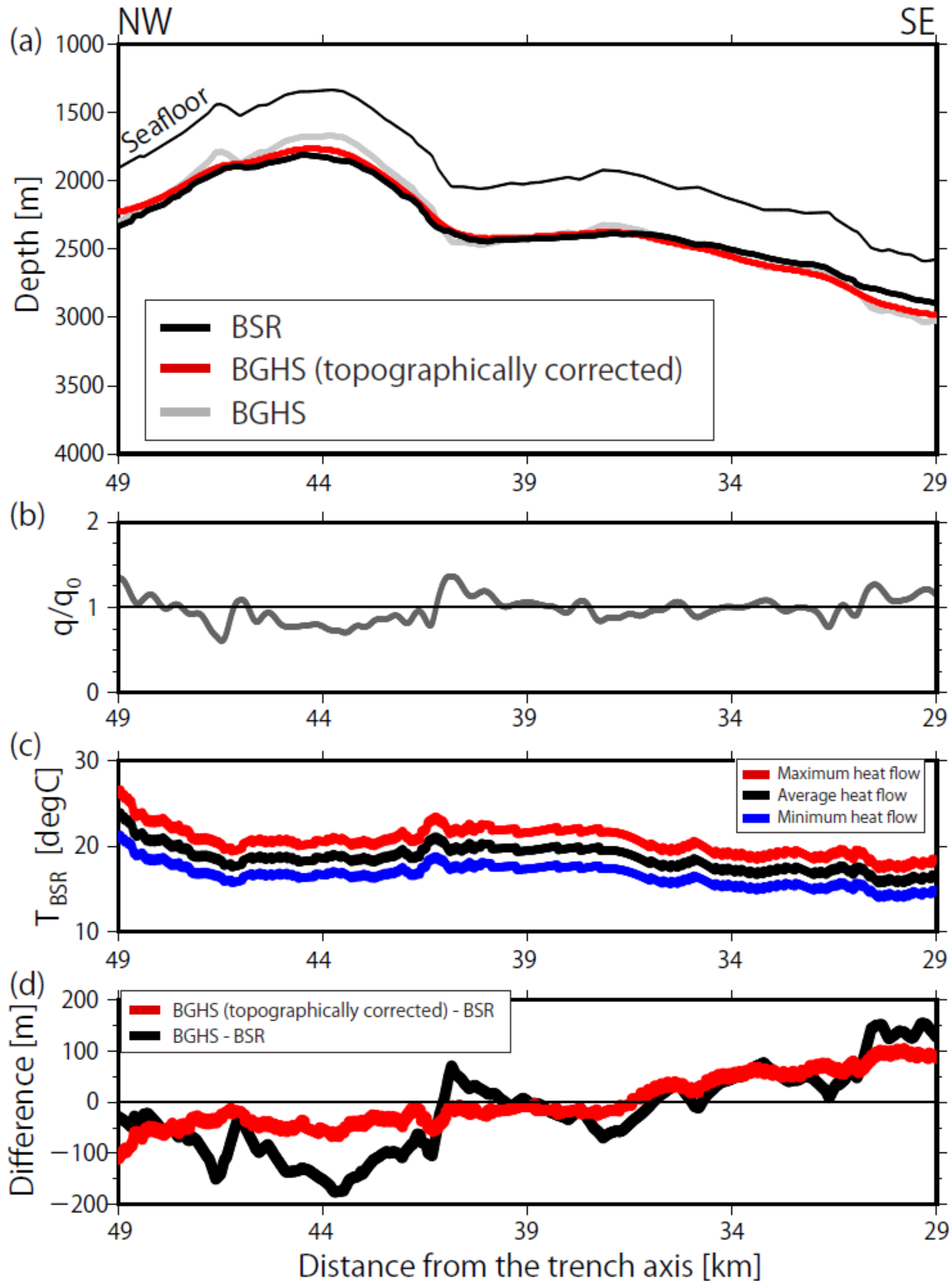

Figure S19. BSR and BGHS in the convex-upward and convex-downward seafloor regions at the location indicated as S19 in Figure S3. (a) Bathymetry with the depths of the observed BSR and the calculated topographically corrected (2-D) and uncorrected (1-D) BGHS. (b) A plot of q/q_0 ratio, where q_0 is the heat flow originating from the deep-seated heat flux and q is estimated heat flow at the seafloor from deep-seated heat flux. (c) Error evaluation of the thermal modeling by comparison to temperature on the BSR (T_{BSR}) using maximum (red line), average (black line), and minimum (blue line) heat flow values. (d) Difference in depths between the observed BSR and calculated BGHS values with and without considering the topographic effect.

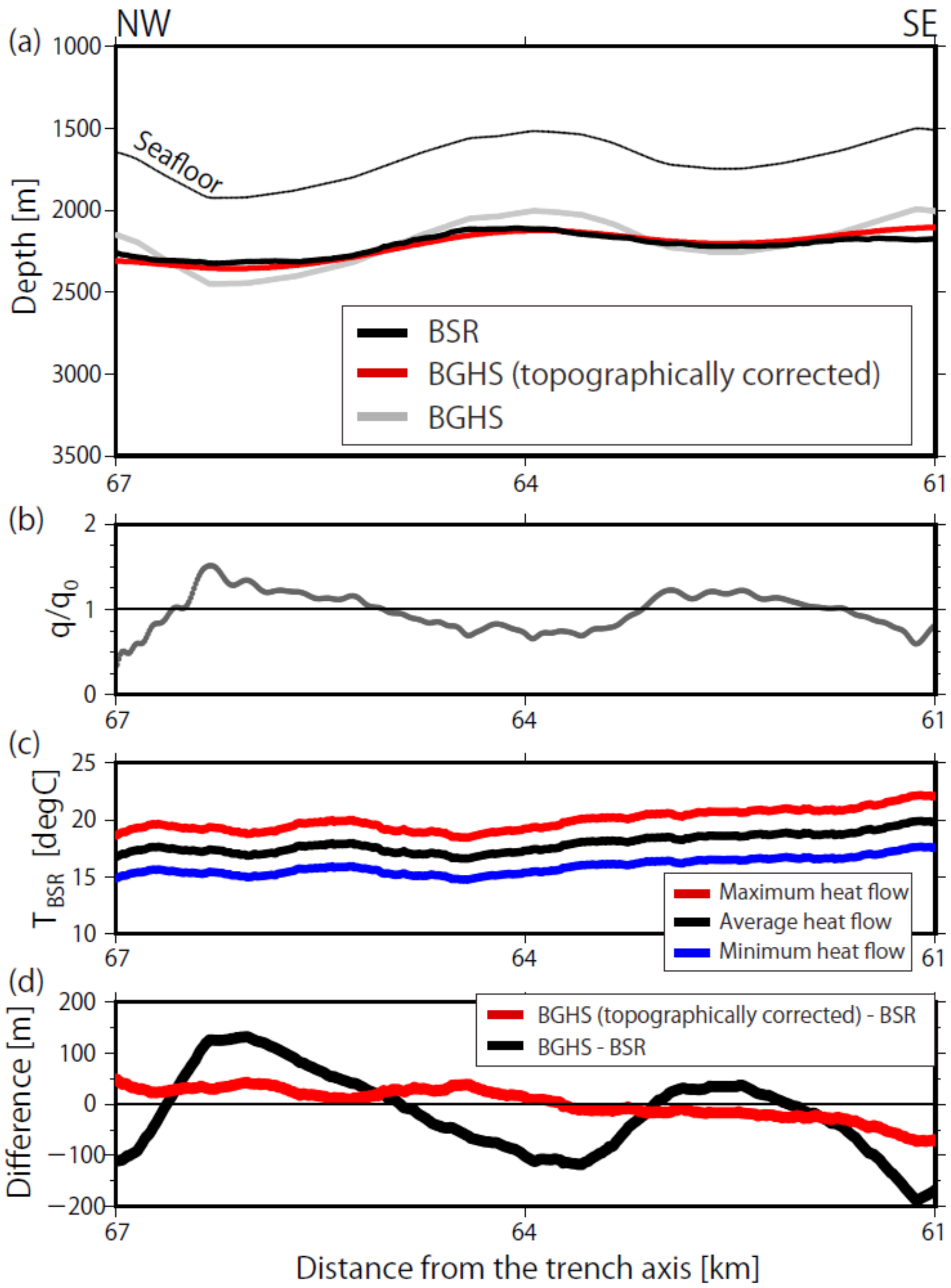

Figure S20. BSR and BGHS in the convex-upward and convex-downward seafloor regions at the location indicated as S20 in Figure S3. (a) Bathymetry with the depths of the observed BSR and the calculated topographically corrected (2-D) and uncorrected (1-D) BGHS. (b) A plot of q/ q_0 ratio, where q_0 is the heat flow originating from the deep-seated heat flux and q is estimated heat flow at the seafloor from deep-seated heat flux. (c) Error evaluation of the thermal modeling by comparison to temperature on the BSR (T_{BSR}) using maximum (red line), average (black line), and minimum (blue line) heat flow values. (d) Difference in depths between the observed BSR and calculated BGHS values with and without considering the topographic effect.

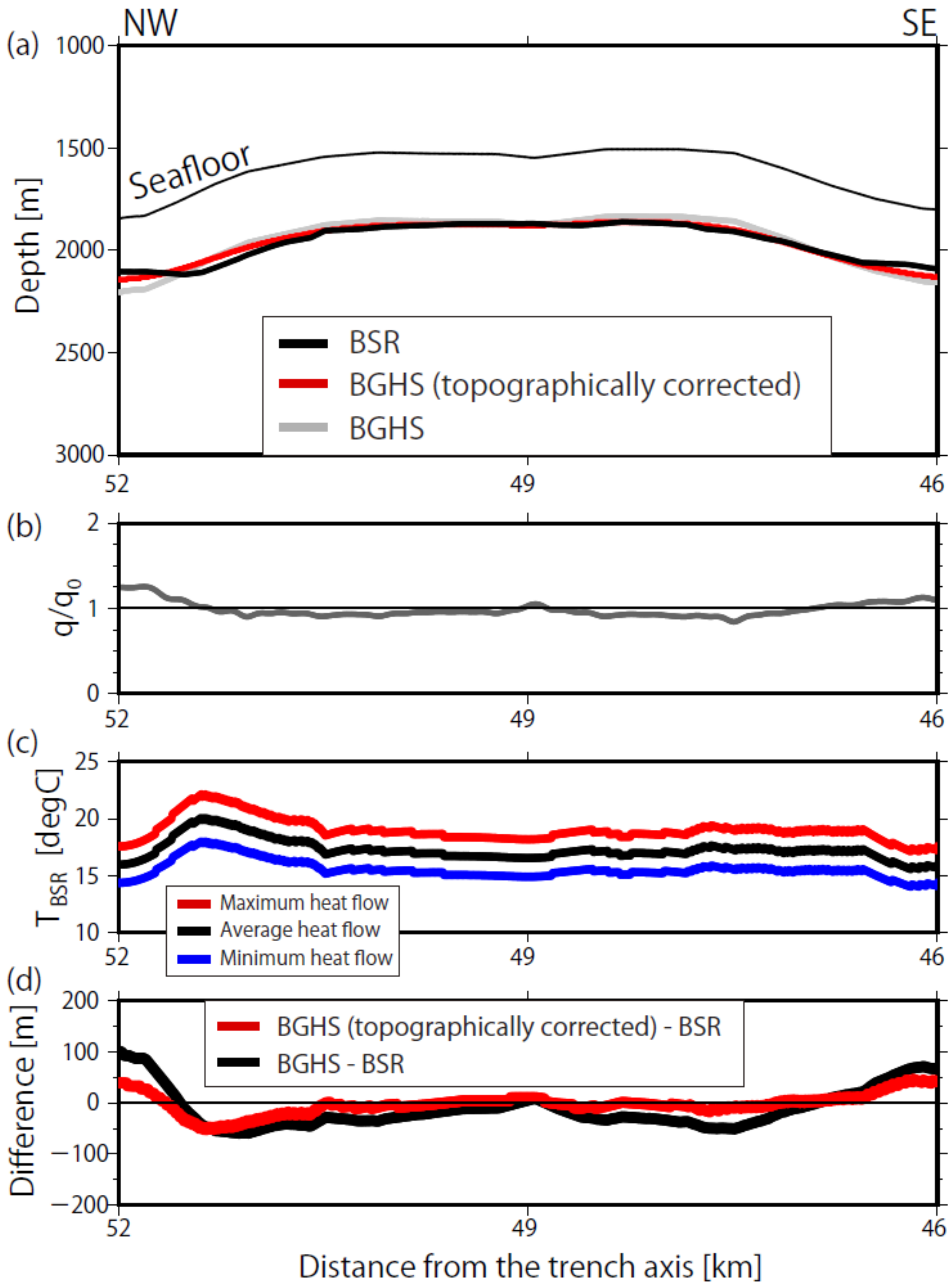

Figure S21. BSR and BGHS in the convex-upward and convex-downward seafloor regions at the location indicated as S21 in Figure S3. (a) Bathymetry with the depths of the observed BSR and the calculated topographically corrected (2-D) and uncorrected (1-D) BGHS. (b) A plot of q/ q_0 ratio, where q_0 is the heat flow originating from the deep-seated heat flux and q is estimated heat flow at the seafloor from deep-seated heat flux. (c) Error evaluation of the thermal modeling by comparison to temperature on the BSR (T_{BSR}) using maximum (red line), average (black line), and minimum (blue line) heat flow values. (d) Difference in depths between the observed BSR and calculated BGHS values with and without considering the topographic effect.

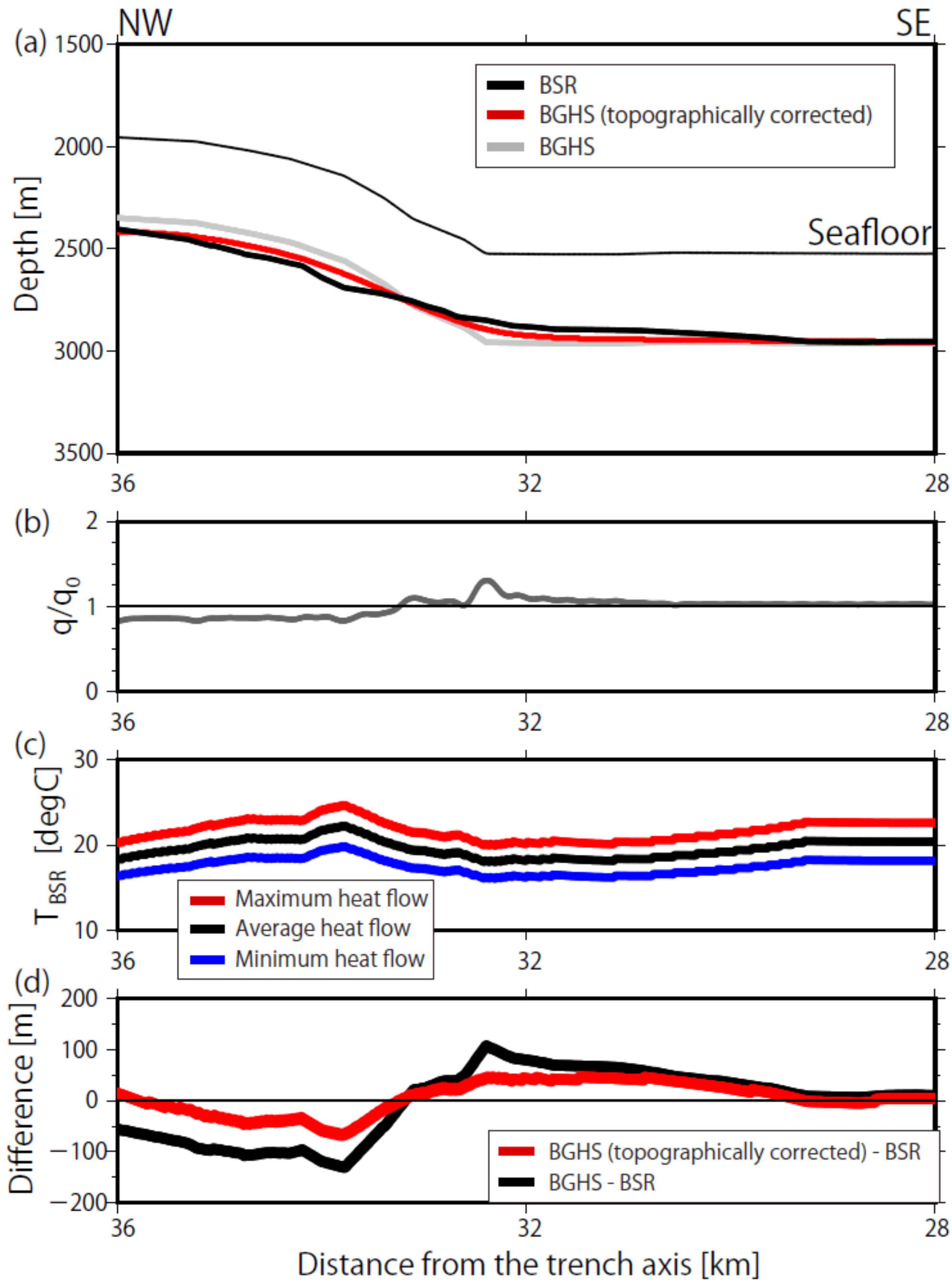

Figure S22. BSR and BGHS in the convex-upward and convex-downward seafloor regions at the location indicated as S22 in Figure S3. (a) Bathymetry with the depths of the observed BSR and the calculated topographically corrected (2-D) and uncorrected (1-D) BGHS. (b) A plot of q/ q_0 ratio, where q_0 is the heat flow originating from the deep-seated heat flux and q is estimated heat flow at the seafloor from deep-seated heat flux. (c) Error evaluation of the thermal modeling by comparison to temperature on the BSR (T_{BSR}) using maximum (red line), average (black line), and minimum (blue line) heat flow values. (d) Difference in depths between the observed BSR and calculated BGHS values with and without considering the topographic effect.

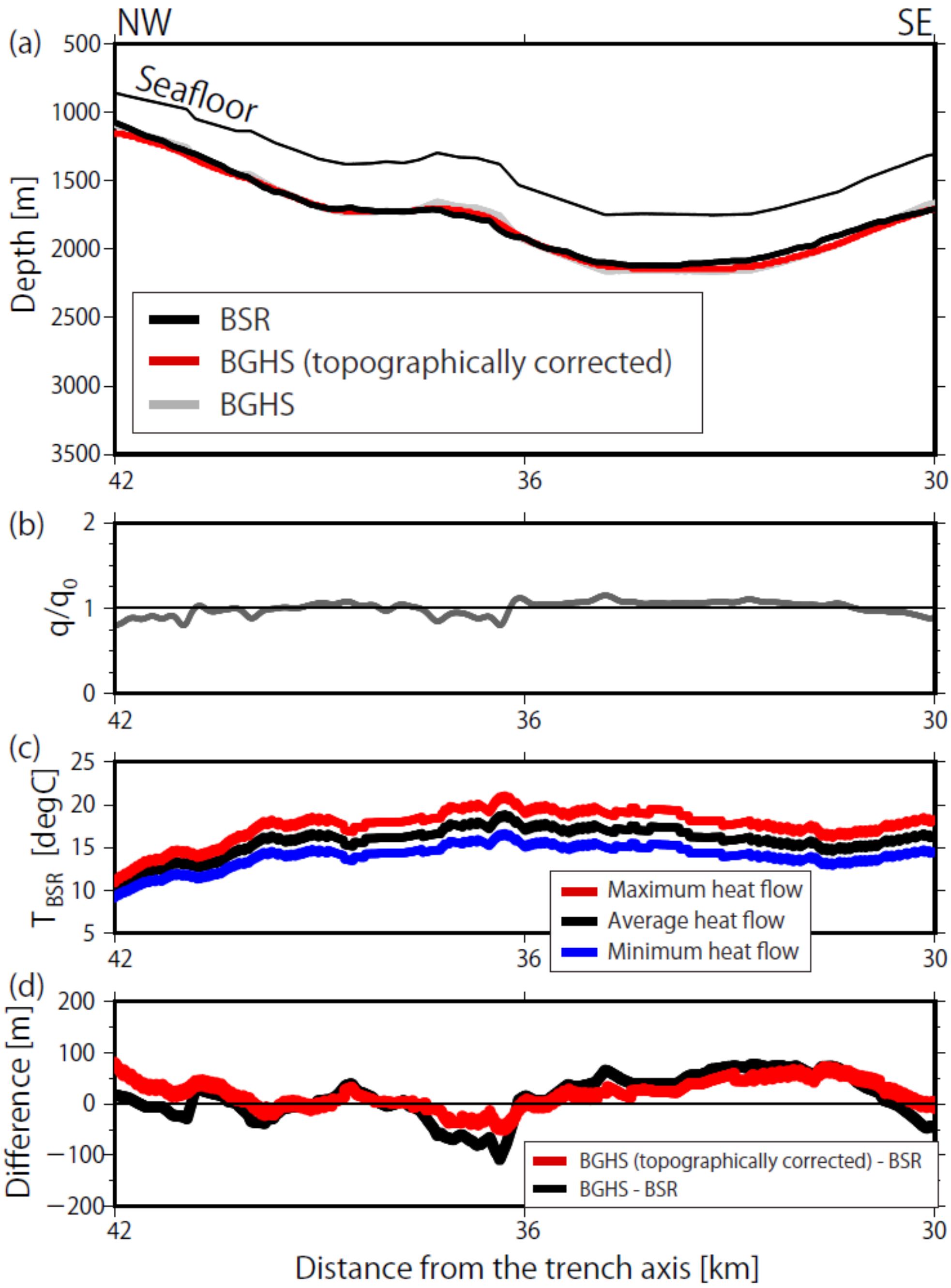

Figure S23. BSR and BGHS in the convex-upward and convex-downward seafloor regions at the location indicated as S23 in Figure S3. (a) Bathymetry with the depths of the observed BSR and the calculated topographically corrected (2-D) and uncorrected (1-D) BGHS. (b) A plot of q/ q_0 ratio, where q_0 is the heat flow originating from the deep-seated heat flux and q is estimated heat flow at the seafloor from deep-seated heat flux. (c) Error evaluation of the thermal modeling by comparison to temperature on the BSR (T_{BSR}) using maximum (red line), average (black line), and minimum (blue line) heat flow values. (d) Difference in depths between the observed BSR and calculated BGHS values with and without considering the topographic effect.

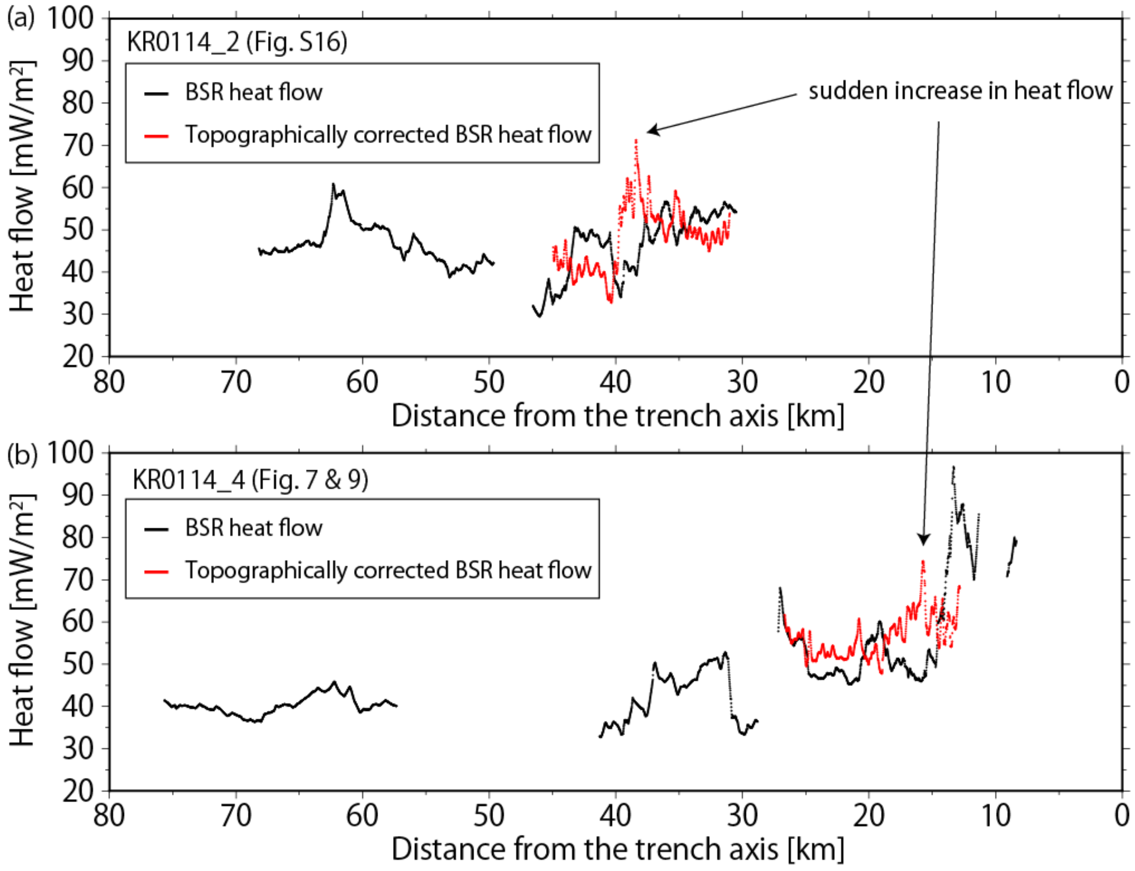

Figure S24. Heat flow profiles at distances from the trench axis. Red lines correspond to the locations of the modeling sections in Figures S16 (a) and 7, 9 (a). (a) A sudden increase in topographically corrected BSR-derived heat flow is confirmed at around 40 km from the trench axis. (b) A sudden increase in topographically corrected BSR-derived heat flow is confirmed at around 15 km from the trench axis.
